# Prevalence of chronic cough in China: a systematic review and meta-analysis

**DOI:** 10.1186/s12890-022-01847-w

**Published:** 2022-02-12

**Authors:** Hanwen Liang, Weiyan Ye, Zhufeng Wang, Jingyi Liang, Fang Yi, Mei Jiang, Kefang Lai

**Affiliations:** 1 National Clinical Research Center for Respiratory Disease, State Key Laboratory of Respiratory Disease, Guangzhou Institute of Respiratory Health, The First Affiliated Hospital of Guangzhou Medical University, Guangzhou Medical University, Guangzhou, Guangdong China; 2 Guangzhou Blood Center, Guangzhou, Guangdong China

**Keywords:** China, Cough, Chronic diseases, Meta-analysis, Prevalence

## Abstract

**Background:**

Individual studies have indicated variable prevalence for chronic cough, but thus far, there has been no systematic report on the prevalence of this condition.

**Methods:**

In this study, we performed a systematic review and meta-analysis by searching databases including PubMed, Cochrane Library, Web of Science, China National Knowledge Infrastructure, Chinese biomedical literature service system, Wanfang Database, and VIP database, for studies on chronic cough in China published before December 28, 2020. A random effects model was used to calculate pooled prevalence estimates with 95% confidence interval [95%CI], weighted by study size.

**Results:**

Fifteen studies with 141,114 community-based adults were included in the study, showing a prevalence of 6.22% (95% CI 5.03–7.41%). And 21 studies with 164,280 community-based children were included, presenting a prevalence of 7.67% (95% CI 6.24–9.11%). In subgroup meta-analyses, the prevalence in adults was 4.38% (95% CI 2.74–6.02%) in southern China and 8.70% (95% CI 6.52–10.88%) in northern China. In the children population, the prevalence in northern China was also higher than in southern China (northern vs. southern: 7.45% with a 95% CI of 5.50–9.41%, vs. 7.86% with a 95% CI of 5.56–10.16%).

**Conclusions:**

Our population-based study provides relatively reliable data on the prevalence of chronic cough in China and may help the development of global strategies for chronic cough management.

**Supplementary Information:**

The online version contains supplementary material available at 10.1186/s12890-022-01847-w.

## Background

Cough is an essential defense mechanism, which prevents the aspiration of excessive respiratory secretions and foreign bodies [1]. However, cough is also one of the most common symptoms and subject of complaints among patients seeking help from respiratory specialists and community outpatient clinics [2]. Chronic cough is defined as a cough that lasts eight weeks or longer in adults, or four weeks or longer in children [3–7], seriously impairs life quality, and results in a heavy social and economic burden [8–10]. Worldwide, more than 10% percent of the adults suffer from chronic cough, and in China, patients with chronic cough account for more than a third of the total patients in respiratory clinics [11, 12].

Previously, chronic cough was considered a concomitant symptom in various diseases, including in asthma, rhinitis, and gastro-esophageal acid reflux disease, and was ignored [13]. However, recent evidence suggests that chronic cough is a clinical syndrome with a distinct and intrinsic pathophysiology, characterized by neuronal hypersensitivity, significant association with a drastic decrease of lung function, and an increase risk of hospitalization [14–16].

Chronic cough has gained increasing attention in recent years and emerged as a serious public health problem. Since the first Cough Guideline launched in 1998, countries have successively issued guidelines to standardize the definition and the treatments of chronic cough [17]. Recent research has focused on risk factors, mechanisms and treatments for chronic cough in China. Yet, the epidemiology of chronic cough, also important for its management, is rapidly changing with the urbanization of China [1, 18–21]. Although a research letter published in 2015 [12] reviewed the global burden of chronic cough, the prevalence of chronic cough in China had not been systematically and independently reported. Considering the role of host–environment interactions, we hypothesized that chronic cough might have distinct characteristics in China. As there are more than 1.4 billion people in China, epidemiological information on chronic cough in this country cannot be ignored, and may contribute to the definition of global strategies for the management of this distressing disease. The whole world, as China, urgently requires updated information on chronic cough prevalence and burden among the general population.

We performed a systematic review of the studies performed on the Chinese population that reported chronic cough prevalence in different regions and over different periods of time. We hypothesized that these data would provide crucial updates regarding chronic cough disease burden in China and bring useful information to plan appropriate strategies for the allocation of healthcare resources. We pooled chronic cough prevalence estimates from different regions and provinces of China and analyzed the prevalence of chronic cough among Chinese adults and children. Understanding the epidemiologic patterns of chronic cough in the Chinese population will lead to a better management of this disease in China and provide data to estimate the burden of chronic cough worldwide.

## Methods

This systematic review was performed in accordance with the Preferred Reporting Items for Systematic Reviews and Meta-Analyses statement (PRISMA) 2020 [22]. Besides, we prospectively submitted the systematic review protocol for registration on PROSPERO (CRD42021247623).

### Search strategies and selection criteria

A systematic search using a combination of keywords including “chronic cough” or “prevalence” and “China,” was performed independently in seven different databases, including PubMed, Cochrane Library, Web of Science, China National Knowledge Infrastructure, Chinese biomedical literature service system, Wanfang Database, and VIP database. The search strategies were drafted independently by H.W. and Z.F. and evaluated according to the inclusion criteria. Disagreements were discussed until a consensus was reached. To minimize missingness, a manual search in the bibliography of reference articles, as well as in previously published relevant reviews was performed. A quality control of the literature search was conducted by M.J. All suitable articles published before the 28th of December 2020 were identified and subsequently catalogued using EndNote X9. All articles published in Chinese or English were included. The detailed search strategy is described in Supporting Information (Additional file 1).

Comprehensive inclusion and exclusion criteria were predefined to facilitate the objective screening of the articles (Table 1). Suitable reports identified by manual search were also included for review. The references of system review and meta-analysis were also reviewed. Two reviewers (H.W. and Z.F.) independently reviewed all reports in accordance with the preset criteria. The outcome of this initial review was then cross checked by the two reviewers. Conflicting opinions and uncertainties were discussed and resolved by reaching a consensus with a third reviewer.

**Table 1:** Inclusion and exclusion criteria for article selection used in the systematic review, according to the PICOS Framework

Component	Inclusions	Exclusion
Population	Community-based or unselected populations of China	1. Reports that focused only on specific sub-groups (e.g., soldiers and patients with occupational diseases) 2. Participants are from studies based on respiratory clinic or focused on a defined disease like bronchitis, COVID-19, influenza virus, mycoplasma pneumoniae infection etc 3. Studies using duplicated samples
Intervention and comparator	Any	Not applicable
Outcome	Studies reporting the prevalence of chronic cough, or data that can be converted into prevalence, such as calculate the prevalence according to the formulation of [ (number of female chronic cough patients + number of male chronic cough patients) / (number of female participants + male participants)] *100%. Studies with chronic cough or other conceptually equivalent terms, such as prolonged cough or persistent cough	1. Studies published neither in English nor Chinese 2. Full text not accessible 3. Studies reported the proportion of chronic cough based on population for medical care
Study design	Observational study, such as cohort study and cross-sectional study	Case reports, case series, comments, conference papers, technical reports, popular science literature, and animal experiments

### Data extraction and quality assessment

A full text review was performed for all selected article and the data were extracted and sorted by two reviewers (H.W. and Z.F.) using independent spreadsheets, into the following variables: first author, publication year, title, region, participants’ demographic characteristics, diagnostic criteria for chronic cough, number of cases, sample size, journal type, and prevalence of chronic cough. When data were missing, the corresponding authors of the concerned articles were contacted to obtain relevant information. For studies containing data from different provinces or age groups, the relevant data were extracted separately according to provinces and age categories (adults ≥ 18 years, or children). The two reviewers (H.W, and J.Y.) assessed independently the quality of the included studies using an 11-item checklist recommended by the Agency for Healthcare Research and Quality (AHRQ) (Additional file 2). An item was scored “0” if it was answered “NO” or “UNCLEAR,” and “1” if it was answered “YES.” According to this scoring, the article quality was defined as follows: low quality = 0–3; moderate quality = 4–7; high quality = 8–11 [23, 24]. If no consensus could be reached between the two reviewers, a third reviewer (W.Y.) was consulted. The grading of recommendations assessment, development, and evaluation (GRADE) algorithm was used to assign quality levels to the meta-analysis evidence. The overall confidence could be judged as ‘‘high,’’ ‘‘moderate,’’ ‘‘low,’’ or ‘‘very low’’ [25].

### Statistical analysis

The pooled prevalence was calculated using the inverse-variance random-effects model or fix-effect model, which was presented as percentage with 95% confidence intervals. Heterogeneity was assessed using the I^2^ statistic. Subgroup analysis as well as heterogeneity regression analysis were performed to determine if the prevalence data was influenced by age, region, AHRQ, diagnosis criteria, year of publication, sample size, prevalence definition, chronic cough definition, and sampling method. Publication bias was assessed by funnel plots and Begg's test. Sensitivity analyses was conducted by plotting the pooled effect size and excluding one study at a time to estimate its individual effect on the results overall (The pooled results was robust, if we removing any particular study not change the pooled effect size or significance of the remaining studies). Stata 14.0 was used for the analysis. The significance level was defined as (two-tailed) P < 0.05.

## Results

### Study selection

**Fig. 1:**
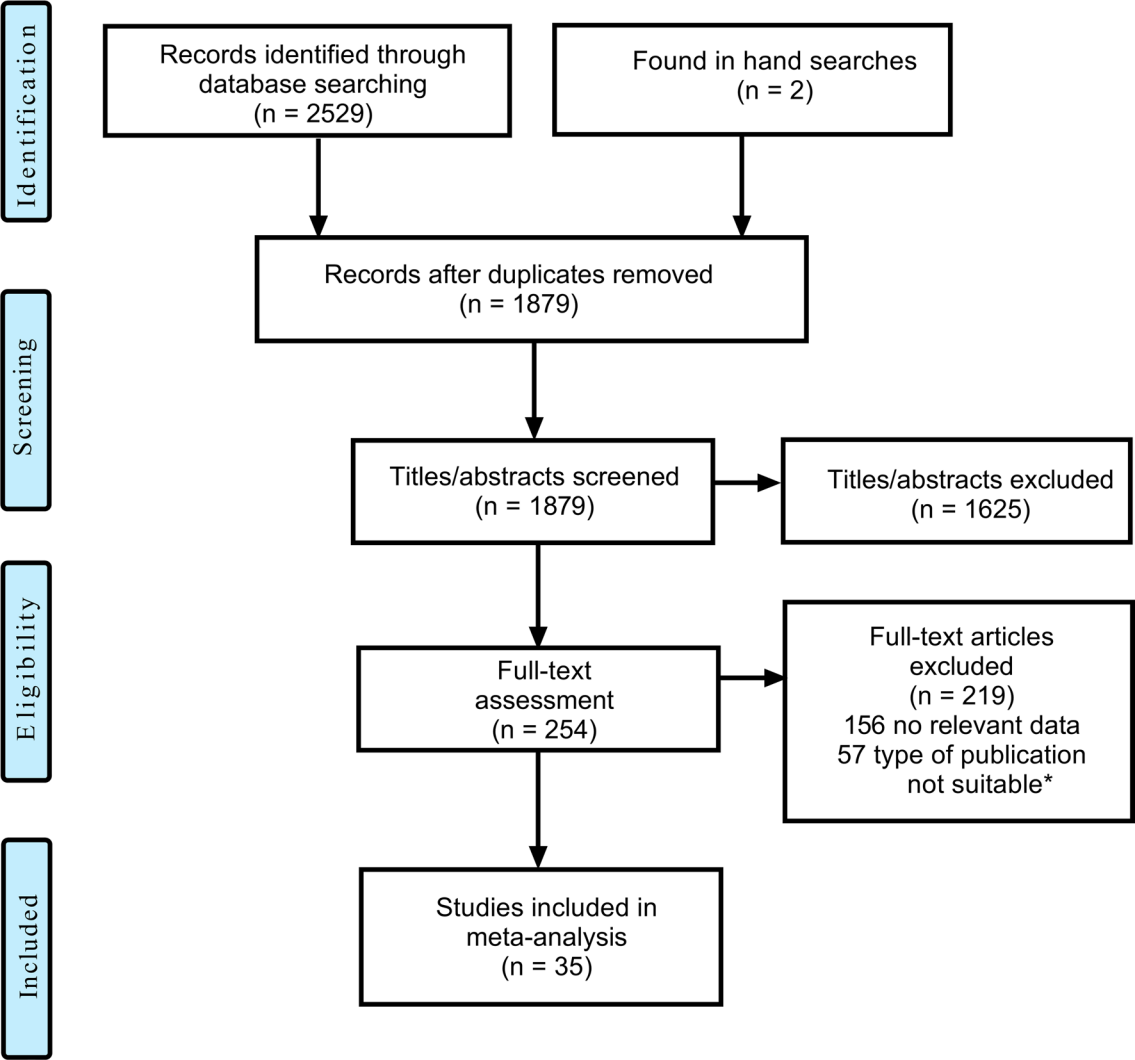
Flow diagram of records identified, excluded and included in review. *Note*: “*” type of publications were reviews, case reports, comments, clinical trials or meta-analysis

The literature search yielded a total of 2531 potentially relevant citations, of which 652 were duplicates, i.e., the investigations were performed in population or subset of population already included. After screening (title, abstract), a total of 254 articles were retained for full-text review. After comprehensive full-text review, 35 articles (21 in Chinese and 14 in English) were finally included, of which 15 involved adults and 21 involved children. Figure 1 and Additional file 3 details the process of studies selection and the reasons of exclusion.

### Quality assessment

All selected articles were assessed for methodological quality. Among the studies reporting the prevalence of chronic cough in adults, two were of high quality [26, 27], and thirteen were of moderate quality [28–40]. Among the studies reporting the prevalence of chronic cough in children, 2 were of high quality [41, 42], and 19 were of moderate quality [36, 43–60]. No articles ranked as low-quality (Additional file 4). For studies in adults and children, the AHRQ score was 5.46 ± 1.46 and 5.05 ± 1.24, respectively. The GRADE evidence of all outcomes was judged as “moderate’’, ‘‘low’’, or ‘‘very low’’. These results are shown in Table 2.

**Table 2:** Principal characteristics of studies in adults included in the meta-analysis

References	City	Diagnostic criteria	Age (y)	Events	Total	Prevalence (%)	Bias score	GRADE^a^	Source of information	Journal classification
Koo et al. [36]	Hongkong	Cough more than 3 months	37.8	18	314	5.7	4	Low	Cluster random sampling	SCI^b^
Lai et al. [30]	Hongkong	Not mentioned	≥ 70	203	2032	10	4	Low	Stratified random sampling	SCI
Zhang et al. [35]	Lanzhou	Cough more than 3 months	18–49	83	1494	5.556	4	Very low	Cluster random sampling	Other
Zhang et al. [35]	Wuhan	Cough more than 3 months	18–49	47	1524	3.08	4	Very low	Cluster random sampling	Other
Zhang et al. [35]	Guangzhou	Cough more than 3 months	18–49	8	1090	0.73	4	Very low	Cluster random sampling	Other
Venners et al. [33]	Anhui	Cough in the morning for three or more months during the winter	≥ 18	73	2525	2.89	4	Moderate	Random samples	SCI
Venners et al. [33]	Beijing	Cough in the morning for three or more months during the winter	≥ 18	92	1184	7.81	4	Moderate	Random samples	SCI
Chen et al. [28]	Guangzhou	Cough more than 3 weeks	21 ± 1	36	1087	3.3	6	Low	Cluster random sampling	The core journal of China
Wilson et al. [37]	Liaoning	Cough more than 3 months	47.7 ± 15.2	729	31,704	2.3	5	Low	Cluster random sampling	SCI
Pan et al. [53]	Guangzhou	Cough more than 8 weeks	20 ± 4	58	2588	2.24	4	Very low	Census	Other
Wang et al. [27]	Beijing	Not mentioned	≥ 18	118	7614	1.55	8	Very low	Cluster random sampling	The core journal of China
Wang [29]	Gansu	Cough more than 3 months, for as much as 2 year	≥ 40	175	728	24.04	5	Very low	Not mentioned	The core journal of China
Li [32]	Shenzhen	Cough more than 3 weeks	≥ 18	53	1468	3.6	5	Low	Cluster random sampling	Other
Yue [26]	Xi’an	Not mentioned	≥ 60	73	758	9.6	8	Low	Cluster random sampling	Other
Hu et al. [38]	Beijing	Cough more than 8 weeks	≥ 35	156	1003	15.6	7	Moderate	Stratified random sampling	SCI
Huang et al. [40]	Foshan	Cough more than 8 weeks, which is the main or only symptom	≥ 18	153	1769	8.65	5	Low	Multi-stage random sampling	The core journal of China
Zhang et al. [39]	Beijing	Cough more than 3 months	≥ 20	1894	26,166	7.2	6	Moderate	Cluster random sampling	SCI
Li [31]	China	Cough more than 3 months	≥ 40	2971	56,066	5.3	7	Moderate	Stratified multi-stage cluster sampling	The core journal of China

^a^GRADE, The Grading of Recommendations Assessment, Development, and Evaluation [25]

^b^SCI, Science Citation Index

### Geographical coverage

The 35 selected studies, 1 nationwide epidemiological investigation and studies from 12 provinces and autonomous regions of China. Several studies covered more than one region. The detail of the geographical coverage is shown in Table 2.

### Characteristics of participants

In total, the 35 studies included 305,394 participants (141,114 adults and 164,280 children), of which 20,177 (6,940 adults and 13,237 children) were patients with chronic cough. The characteristics of the participants are presented in Tables 2, 3 and Additional file 5.

**Table 3:** Principal characteristics of studies in children included in the meta-analysis

References	City	Diagnostic criteria	Age (y)	Events	Total	Prevalence (%)	Bias score	GRADEa	Source of information	Journal classification
Koo et al. [36]	Hongkong	Cough more than 3 months	10.1	22	314	7.00	4	Low	Cluster random sampling	SCI^b^
Xi et al. [54]	Liaoning	Cough more than 3 months	School-age children	549	15,233	3.604	4	Low	Cluster random sampling	Other
Zhang et al. [56]	Guangzhou	Coughed for at least 1 month per year either with or apart from colds	5.4–16.2	166	2216	7.49	4	Low	Cluster random sampling	SCI
Zhang et al. [56]	Wuhan	Coughed for at least 1 month per year either with or apart from colds	5.4–16.2	211	2307	9.17	4	Low	Cluster random sampling	SCI
Zhang et al. [56]	Lanzhou	Coughed for at least 1 month per year either with or apart from colds	5.4–16.2	157	1438	10.94	4	Low	Cluster random sampling	SCI
Zhang et al. [56]	Chongqing	Coughed for at least 1 month per year either with or apart from colds	5.4–16.2	101	1431	7.06	4	Low	Cluster random sampling	SCI
Xi et al. [55]	Benxi	Cough more than 3 months	School-age children	216	5404	3.997	4	Low	Cluster random sampling	The core journal of China
Cai and Luo [43]	Liaoning	Cough more than 4 weeks	0–14	329	9947	3.31	4	Very low	Cluster random sampling	The core journal of China
Dong et al. [46]	Liaoning	Cough more than 4 days per week for as much as 3 months of the year either with or apart from colds	School-age children, toddler	1480	14,556	10.17	6	Low	Cluster random sampling	Other
Salo et al. [42]	Wuhan	Cough almost every day in the absence of colds during the past 12 months	15.2 ± 0.6	176	4146	4.30	8	Moderate	Cluster random sampling	SCI
Liu et al. [51]	Benxi	Not mentioned	School-age children, toddler	276	2318	11.89	4	Low	Cluster random sampling	The core journal of China
Dong et al. [59]	Liaoning	Cough more than 4 days per week for as much as 3 months of the year	1–13	1347	14,729	9.15	5	Low	Stratified random sampling	SCI
Wu [47]	Shanghai	Cough more than 3 weeks	4–17	519	6551	7.92	4	Low	Cluster random sampling	Other
Niu et al. [52]	Shanghai	Cough on most days (S4 days per week) for as long as 3 months of the year, either together with or separately from cold	4–17	565	6551	8.60	5	Low	Stratified random sampling	The core journal of China
Pan et al. [53]	Liaoning	Cough on most days (S4 days per week) for as long as 3 months of the year, either together with or separately from cold	3–12	1123	11,860	9.47	4	Moderate	Cluster random sampling	SCI
Zhang et al. [50]	Zhongshan	Cough more than 4 weeks	2–12	260	3947	6.587	5	Low	Cluster random sampling	The core journal of China
Gao [48]	Wenzhou	Cough more than 4 weeks	0–14	1544	5843	26.42	5	Very low	Cluster random sampling	Other
Huang et al. [41]	Zhongshan	Cough more than 4 weeks	3–14	889	15,763	5.64	8	Moderate	Cluster random sampling	The core journal of China
Li et al. [58]	Lanzhou	Cough more than 4 days per week for as much as 3 months of the year	8–13	15	929	1.60	6	Very low	Cluster random sampling	The core journal of China
Gao et al. [45]	Hongkong	Not mentioned	8–10	104	2203	4.72	6	Low	Cluster random sampling	SCI
Wang et al. [60]	Liaoning	Cough more than 4 days per week for as much as 3 months of the year	2–14	2846	30,056	9.47	5	Low	Stratified random sampling	SCI
Zhu et al. [57]	Beijing	Not mentioned	5–11	197	4241	4.65	5	Low	Cluster random sampling	The core journal of China
Fan et al. [44]	Chongqing	Not mentioned	8–10	28	695	4.03	6	Very low	Cluster random sampling	The core journal of China
Li et al. [49]	Hebei	Cough more than 4 weeks	2–12	117	1602	7.30	4	Low	Random sampling	Other

^a^GRADE, The Grading of Recommendations Assessment, Development, and Evaluation [25]

^b^SCI, Science Citation Index

### Prevalence

**Fig. 2:**
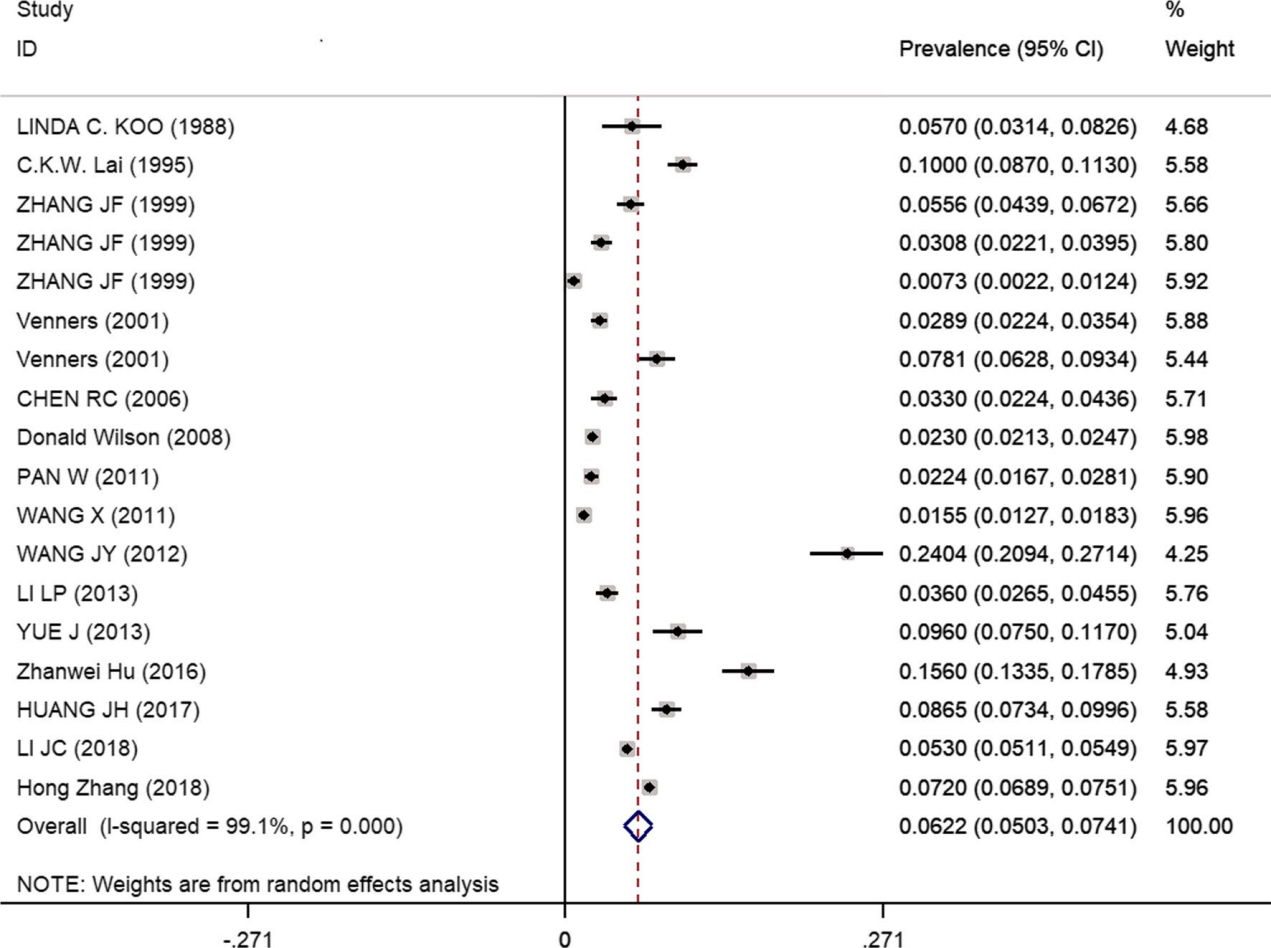
Pooled chronic cough prevalence of adults in China. *CI* confidence intervals

**Fig. 3:**
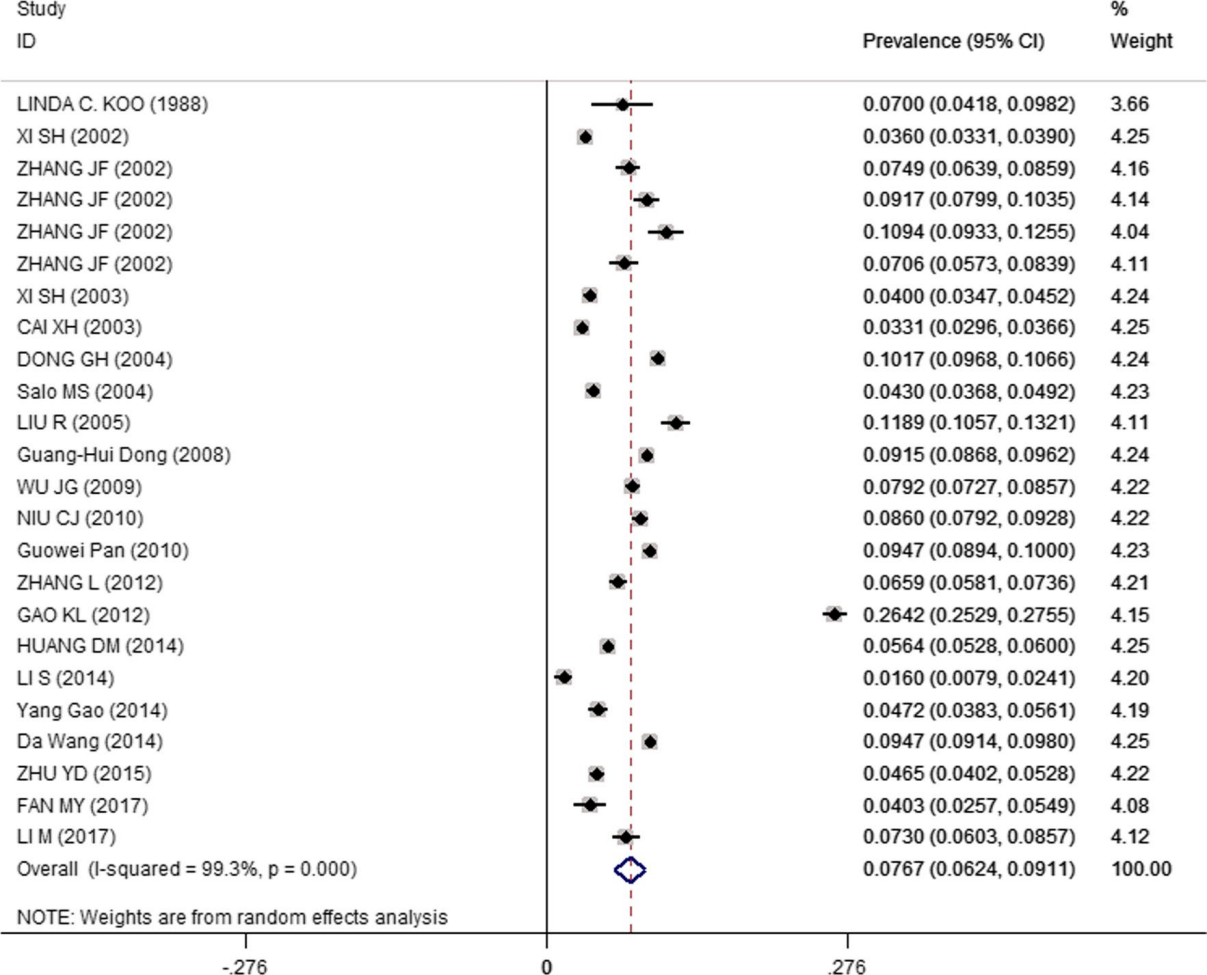
Pooled chronic cough prevalence of children in China. *CI* confidence intervals

**Fig. 4:**
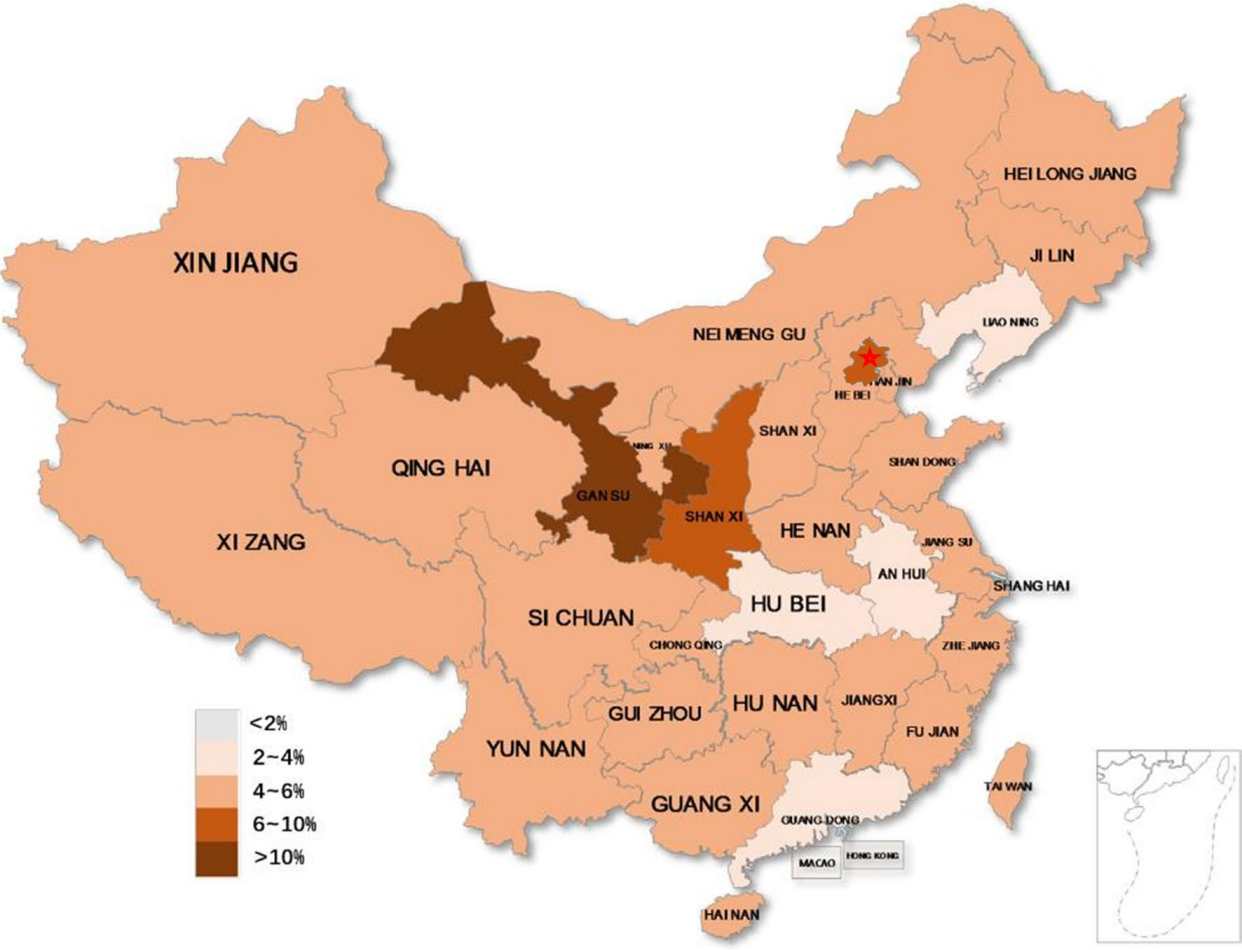
Distribution of adults with chronic cough across Mainland China. *Note*: Red star in the map represents Beijing City. The map was developed in XL Toolbox NG by ourselves, without the conflict of copyright

The overall pooled prevalence of chronic cough was 6.22% (95% CI 5.03–7.41%) in adults and 7.67% (95% CI 6.24–9.11%) in children. There was significant heterogeneity between the studies that reported the prevalence of chronic cough in adults (I^2^ = 99.1%, P < 0.001) and children (I^2^ = 99.3%, P < 0.001) (Figs. 2, 3, 4 and Additional file 6: Fig. S1). A subsequent meta regression analysis to explore the source of heterogeneity and the results showed that the year of publication, sample size, diagnostic definition, classification of prevalence, and region were not associated with heterogeneity between studies (adults: adjusted R^2^: –8.31%, P = 0.581; children: adjusted R^2^: –27.36%, P = 0.988).

### Subgroup analyses

We ran separate meta-analyses on adult studies for subgroup effects by region, diagnostic criteria, AHQR, age, sample size, population sampling method, prevalence definition and year of publication, using the random effects model. The prevalence of chronic cough was 4.38% (95% CI 2.74–6.02%) in southern China, and 8.70% (95% CI 6.52–10.88%) in northern China (Additional file 6: Fig. S2). The pooled prevalence, according to the diagnostic criteria: “cough lasting for more than three weeks”, “cough lasting for more than eight weeks”, and “cough lasting for more than three months”, was 3.47% (95% CI 2.76–4.18%), 8.76% (95% CI 1.82–15.69%) and 6.14% (95% CI 4.32–7.96%), respectively (Additional file 6: Fig. S3). According to the update of the Chinese Guideline for Cough, the included studies were divided into four periods of time (1988–2004; 2005–2009; 2010–2014; and 2015–2020). Compared with the other three periods, the prevalence during 2005–2009 was dramatically low (2.66%, 95% CI 1.72–3.60%) (Additional file 6: Fig. S4). The prevalence of older adults (9.89%; 95%CI: 8.78–11.00%) was higher than non-elderly adults (3.25%; 95%CI: 1.85–4.64%) significantly (Additional file 6: Fig. S5). The prevalence of random sampling is 5.63% (95%CI: 4.40–6.85%). The prevalence of the census-based study and the study without mentioning sampling are 2.24% (95%CI: 1.67–2.81%) and 24.04% (95%CI: 20.94–27.14%), respectively. Compared with the prevalence of all eligible studies, those of random sampling is slightly lower (Additional file 6: Fig. S6). The analyses considering other subgroups are shown in Additional file 6: Figs. S7–S10.

Further, a separate meta-analysis, using the random effects model, was performed on the studies with children according to same geographical subgrouping as for the studies with adults. In southern China, 7.45% (95% CI 5.50–9.41%) of the children presented with chronic cough, against 7.86% (95% CI 5.56–10.16%) in northern China (Additional file 6: Fig. S11). The pooled prevalence, according to the diagnostic criteria “cough lasting for more than four weeks”, “cough lasting for more than three months”, and “coughing more than four days per week during three months”, was respectively of 9.78% (95% CI 4.98–14.58%), 3.96% (95% CI 3.27–4.65%), and 8.10% (95% CI 6.35–9.85%) (Additional file 6: Fig. S12). When considering the different periods of publication, the pooled prevalence during the first period (1988–2004) was 6.65% (95% CI 4.90–8.40%), and sharply increased to 9.53% (95% CI 7.93–11.13%) during the second period (2005–2009). From the second to the fourth period, the prevalence of chronic cough in children showed a decreasing trend (Additional file 6: Fig. S13). The analyses considering other subgroups are shown in Supplementary Materials (Additional file 6: Figs. S14–S17).

### Bias and sensitivity analyses

Bias tests were performed on both the adult- and children-related studies. The funnel plots and by the Begg's test (z = 1.29, P = 0.198) indicated that there was no publication bias in the included studies on adults (Additional file 6: Fig. S18). We performed the sensitivity analyses by removing individual studies, which did not change the direction or significance of the pooled results, revealed that the pooled prevalence for the adult population was robust (Additional file 6: Figs. S19-S21). Similarly, no publication bias existed in the included studies involving children (Additional file 6: Fig. S22) (Begg's test: z = 1.22, P = 0.224). The sensitivity analyses revealed that the pooled prevalence for the children population was also robust (Additional file 6: Fig. S23).

## Discussion

Our meta-analysis showed that the prevalence of chronic cough in China is 7.11% (6.22% in adults and 7.67% in children), suggesting that more than 90 million individuals in China are suffering from this condition [61] (Additional file 6: Fig. S24). Chronic cough continues being a major public health issue in China and worldwide, and should not be ignored. Although a meta-analysis reported a global prevalence of chronic cough of 9.6% (95% CI 7.6–11.7%), this previous study did not analyze the prevalence in China separately [12]. Considering the role of host-environment interactions in coughing, we thought that the prevalence of chronic cough in China might be different from the global prevalence. Therefore, data on global prevalence might be of limited value for the management of chronic cough in China.

Cough is one of the important reflexes of the respiratory system, and its mechanism is complex and has not been fully elucidated to date. Activation of TRPA1 and TRPV1 channels on airway sensory nerve terminals and airway inflammation involving various cytokines such as prostaglandins, IFN—γ, and ATP can induce cough [62–64]. Dysregulation of central regulation is also involved in the development of cough [65–67]. Drugs targeting on relevant channels are being developed. The antagonists of TRPA1, TRPV1 and the voltage-gated sodium channel (NaV1.7) showed poor effect [68–70], while P2X3 antagonists, neuropeptide receptor antagonists (NK-1 receptor antagonists) are promising, but further clinical trials are still needed [71, 72].

We found that the prevalence of chronic cough in China was lower than the global prevalence. [12] However, the research of global prevalence conducted by Song et al. [12] included studies covering on a shorter and earlier period from 1980 to 2013, while we included studies covering a period between 1988 and 2020. The number of participants enrolled through studies performed after 2013 represented a considerable proportion of the total participants. Our study also showed that the prevalence of chronic cough in children decreased during the past five years, compared with that between 2005 and 2014. The first guideline for cough in China was published in 2005 [73] and updated successively in 2009 [74] and 2015 [75]. We speculate that these updates, combined with a better understanding of the mechanisms of chronic cough, might have contributed to better management of chronic cough and lower prevalence in China. In addition, approximately half (48.89%) of the studies included in the meta-analysis performed by Song et al. [12] were focused on European populations, which presented a high prevalence (12.7%; 95% CI 10.4–15.2%) and might have biased the global prevalence. A nationwide investigation in China focused on adults over 40 years reported a prevalence of 5.3% for chronic cough, which is closer to the prevalence found for adults in our studies [31].

In adults, there were regional differences in the chronic cough prevalence within China, which was higher in northern than in southern China. To some degree, the prevalence of chronic cough was associated with the level of urbanization and severity of environmental pollution [75]. It has been reported that the concentration of air pollutants was higher in northern China than in southern China [76, 77]. Number of research reported that the air pollution is an important risk factor for chronic cough [19, 21, 32, 40, 49, 50]. The evidence mentioned above imply that environmental factors may account for the regional variation in the prevalence of chronic cough. Several studies showed that the prevalence of chronic cough in urban areas was higher than in countryside, suggesting that urbanization might also contribute to the regional variability [40, 50]. However, we were unable to examine this relationship because relevant information in the included studies was sparse.

The variability of the definitions of chronic cough between studies might have introduced some heterogeneity and affected the calculation of prevalence. However, subgroup analyses taking the definitions of chronic cough into account dramatically showed that the most stringent temporal definition, for both adults and children (adults: more than 3 months; children: more than 4 days per week for as much as 3 months of the year) did not lead the lowest prevalence. Heterogeneity of prevalence still existed in all subgroups, without significant decrease, implying that other factors may contribute to this heterogeneity. The first Chinese Guideline for Cough was published in 2005 [73], using as temporal definition “more than or equal to 8 weeks for adults”. However, this still varied between studies published after edition, implying that the guideline was not strictly followed by Chinese researchers and clinicians. The same problem was also found in studies related to the child populations. In our opinion, poor compliance to this guideline could not only lead to misdiagnosis, but also create difficulties for the management of chronic cough in China. In the future, more effort should be made to reach a consensus definition and promote the guideline across China.

Differences between chronic cough in adults and children have been widely reported. The etiology of pediatric chronic cough included asthma, postinfectious cough, bronchiectasis, airway malacia, and protracted bacterial bronchitis. In contrast, common causes of chronic cough in adults are gastroesophageal reflux, asthma, and upper airway syndrome (e.g., post-nasal drip) [78]. In our studies, we found that the pooled prevalence of chronic cough in adults was lower than that of children (adults vs. children: 6.22% [95% CI 5.03–7.41%] vs. 7.67% [95% CI 6.24–9.11%]). We supposed that the mechanisms described thereafter might contribute to the higher prevalence in children. Cough serves to prevent the lung from inhaling noxious agents and clean the airway of unwanted secretions [79]. In adults, mucus glands constitute about 12% of the bronchial wall, whereas in children, this area is approximately of 17% [80], resulting in greater mucus secretion during childhood. In addition, cough is also a neuromuscular phenomenon involving various respiratory and extra respiratory muscles, and activation by multiple peripheral (e.g., vagal nerves) and central neural circuits of cough reflex [81]. Exposure of the airways to noxious agents may cause more damage in children than in adults. Moreover, chronic cough in infants and children may lead to a greater vulnerability to infections due to irreversible gene upregulation in the vagal afferent nerves by airway inflammation triggered by allergens or viral infections [64, 82].

The present study has several strengths. Firstly, many studies focused on patients with chronic cough in specialized care centers or in general practitioner's office and reported the proportion of chronic cough based on population for medical care. However, the proportion only based on population for medical care would overestimate the prevalence of general population. To our knowledge, our study is the first comprehensive review on the prevalence of chronic cough in China. In addition, we included only population-based data, which limited selection bias. Besides, the latest versions of the PRISMA and PROSPERO protocol were strictly observed, which makes our results more reliable.

Yet, we acknowledge several limitations in our study. First, the definitions of chronic cough were heterogeneous [83], and thus, the estimation of the prevalence might be biased. Second, our studies only covered 12 provinces or autonomous regions, which might have skewed the prevalence estimation for the whole China. Third, significant heterogeneity exists in our study. Despite our effort, the source of heterogeneity could not be identified because of the limited information in the primary studies. The effect of sex on the prevalence of chronic cough was controversial. Difference of prevalence between male and female could not be explored because of limited information in the included studies. Likewise, ethnic background was not emphasized in the included studies, for the relevant information was limited. However, further research focusing on the population of ethnic would be valuable. Finally, recall bias cannot be avoided in our study.

Although limitations exist, the present meta-analysis provides relatively robust results of the prevalence of chronic cough. The differences between northern and southern China suggest that the prevalence of chronic cough might be influenced by environmental factors. The methodological inconsistencies in the studies of chronic cough in China suggest that there is an urgent need for promoting the corresponding guidelines across China and standardizing the definition of chronic cough. What’s more, although the included studies were published before the COVID-19 pandemic, there is insufficient evidence to consider that the prevalence of chronic cough has significantly changed during the COVID-19 pandemic in China [84]. Hence, our data are still applicable now. Besides, because of the widespread fear of cough in the community, it’s significant to call for more social and academic attention to the impact and burden of cough in patients with chronic cough in this pandemic conditions.

## Conclusions

This systematic review and meta-analysis provided relatively reliable data on the prevalence of chronic cough in China (6.22% in adults and 7.67% in children), which may help developing global strategies for chronic cough management.

## Supplementary Information

**Additional file 1.** Search strategies.

**Additional file 2.** Cross-sectional/prevalence study quality.

**Additional file 3.** Exclusion with reasons.

**Additional file 4.** Quality assessment of the included articles according to scale of Agency for Healthcare Research and Quality.

**Additional file 5.** Methodology of studies included in the meta-analysis.

**Additional file 6.** **Fig. S1.** Distribution of children with chronic cough across Mainland China. NOTE: Red star in the map represents Beijing City. The map was developed in XL Toolbox NG by ourselves, without the conflict of copyright. **Fig. S2.** Pooled chronic cough prevalence of adults stratified by region. Abbreviations: CI, confidence intervals. NOTE: The three author labels of ZHANG JF 1999 are from the same literature, and the two author labels of Venners 2001 are from the same literature. **Fig. S3.** Pooled chronic cough prevalence of adults stratified by diagnostic criteria. Abbreviations: CI, confidence intervals. NOTE: The three author labels of ZHANG JF 1999 are from the same literature, and the two author labels of Venners 2001 are from the same literature. **Fig. S4.** Pooled chronic cough prevalence of adults stratified by year of publication. Abbreviations: CI, confidence intervals. NOTE: The three author labels of ZHANG JF 1999 are from the same literature, and the two author labels of Venners 2001 are from the same literature. **Fig. S5.** Pooled chronic cough prevalence of adults stratified by age. Abbreviations: CI, confidence intervals. NOTE: The three author labels of ZHANG JF 1999 are from the same literature, and the two author labels of Venners 2001 are from the same literature. **Fig. S6.** Pooled chronic cough prevalence of adults stratified by sampling methods. Abbreviations: CI, confidence intervals. NOTE: The three author labels of ZHANG JF 1999 are from the same literature, and the two author labels of Venners 2001 are from the same literature. **Fig. S7.** Pooled chronic cough prevalence of adults stratified by sample size. Abbreviations: CI, confidence intervals; ES, Effect Size. NOTE: The three author labels of ZHANG JF 1999 are from the same literature, and the two author labels of Venners 2001 are from the same literature. **Fig. S8.** Pooled chronic cough prevalence of adults stratified by prevalence definitions. Abbreviations: CI, confidence intervals; ES, Effect Size. NOTE: The three author labels of ZHANG JF 1999 are from the same literature, and the two author labels of Venners 2001 are from the same literature. **Fig. S9.** Pooled chronic cough prevalence of adults stratified by chronic cough definitions. Abbreviations: CI, confidence intervals; ES, Effect Size. NOTE: The three author labels of ZHANG JF 1999 are from the same literature, and the two author labels of Venners 2001 are from the same literature. **Fig. S10.** Pooled chronic cough prevalence of adults stratified by quality of articles assessed by AHRQ. Abbreviations: CI, confidence intervals; ES, Effect Size. NOTE: The three author labels of ZHANG JF 1999 are from the same literature, and the two author labels of Venners 2001 are from the same literature. **Fig. S11.** Pooled chronic cough prevalence of children stratified by region. Abbreviations: CI, confidence intervals. NOTE: The four author labels of ZHANG JF 2002 are from the same literature. **Fig. S12.** Pooled chronic cough prevalence of children stratified by diagnostic criteria. Abbreviations: CI, confidence intervals. NOTE: The four author labels of ZHANG JF 2002 are from the same literature. **Fig. S13.** Pooled chronic cough prevalence of children stratified by year of publication. Abbreviations: CI, confidence intervals. NOTE: The four author labels of ZHANG JF 2002 are from the same literature. **Fig. S14.** Pooled chronic cough prevalence of children stratified by sample size. Abbreviations: CI, confidence intervals. NOTE: The four author labels of ZHANG JF 2002 are from the same literature. **Fig. S15.** Pooled chronic cough prevalence of children stratified by chronic cough definitions. Abbreviations: CI, confidence intervals; ES, Effect Size. NOTE: The four author labels of ZHANG JF 2002 are from the same literature. **Fig. S16.** Pooled chronic cough prevalence of children stratified by quality of articles assessed by AHRQ. Abbreviations: CI, confidence intervals. NOTE: The four author labels of ZHANG JF 2002 are from the same literature. **Fig. S17.** Pooled chronic cough prevalence of children stratified by prevalence definitions. Abbreviations: CI, confidence intervals. NOTE: The four author labels of ZHANG JF 2002 are from the same literature. **Fig. S18.** Funnel plot for prevalence in studies of adults for chronic cough. **Fig. S19.** Sensitivity analysis for prevalence in studies of adults for chronic cough. Abbreviations: CI, confidence intervals. NOTE: The three author labels of ZHANG JF 1999 are from the same literature, and the two author labels of Venners 2001 are from the same literature. **Fig. S20.** The prevalence of chronic cough in adults after exclusion of the nationwide study (Li JC 2018). Abbreviations: CI, confidence intervals. NOTE: The three author labels of ZHANG JF 1999 are from the same literature, and the two author labels of Venners 2001 are from the same literature. **Fig. S21.** The prevalence of chronic cough in adults after exclusion of the low prevalence study (ZHANG JF 1999). Abbreviations: CI, confidence intervals. NOTE: The two author labels of ZHANG JF 1999 are from the same literature, and the two author labels of Venners 2001 are from the same literature. **Fig. S22.** Funnel plot for prevalence in studies of children for chronic cough. **Fig. S23.** Sensitivity analysis for prevalence in studies of children for chronic cough. Abbreviations: CI, confidence intervals. NOTE: The four author labels of ZHANG JF 2002 are from the same literature. **Fig. S24.** Pooled prevalence of chronic cough in China (including adults and children). Abbreviations: CI, confidence intervals. NOTE: The three author labels of ZHANG JF 1999 are from the same literature, the two author labels of Venners 2001 are from the same literature, and the four author labels of ZHANG JF 2002 are from the same literature.

## Data Availability

Not applicable.

## Abbreviations

AHRQ: Agency for Healthcare Research and Quality
CI: Confidence intervals
ES: Effect size
GRADE: The Grading of Recommendations Assessment, Development, and Evaluation
PRISMA: Preferred Reporting Items for Systematic reviews and Meta-Analyses
SCI: Science Citation Index

## References

[1] Spanevello A, Beghé B, Visca D, Fabbri LM, Papi A (2020). Chronic cough in adults. Eur J Intern Med.

[2] Morice AH (2002). Epidemiology of cough. Pulm Pharmacol Ther.

[3] Mukae H, Kaneko T, Obase Y, Shinkai M, Katsunuma T, Takeyama K (2021). The Japanese respiratory society guidelines for the management of cough and sputum (digest edition). Respir Investig.

[4] Chang AB, Oppenheimer JJ, Irwin RS, Panel CEC (2020). Managing chronic cough as a symptom in children and management algorithms: CHEST guideline and expert panel report. Chest.

[5] Lee KK, Davenport PW, Smith JA, Irwin RS, McGarvey L, Mazzone SB (2021). Global physiology and pathophysiology of cough: part 1: cough phenomenology—CHEST guideline and expert panel report. Chest.

[6] Morice AH, Millqvist E, Bieksiene K, Birring SS, Dicpinigaitis P, Domingo Ribas C (2020). ERS guidelines on the diagnosis and treatment of chronic cough in adults and children. Eur Respir J.

[7] Lai K, Shen H, Zhou X, Qiu Z, Cai S, Huang K (2018). Clinical practice guidelines for diagnosis and management of Cough-Chinese Thoracic Society (CTS) Asthma Consortium. J Thorac Dis.

[8] Brooks SM (2011). Perspective on the human cough reflex. Cough (London, England).

[9] Fan B, Chen W (2011). Epidemiological survey on female urinary incontinence in urban areas in Guangzhou. Matern Child Health Care China.

[10] Won HK, Lee JH, An J, Sohn KH, Kang MG, Kang SY (2020). Impact of chronic cough on health-related quality of life in the Korean adult general population: the Korean National Health and Nutrition Examination Survey 2010–2016. Allergy Asthma Immunol Res.

[11] Song WJ, Chang YS, Faruqi S, Kang MK, Kim JY, Kang MG (2016). Defining chronic cough: a systematic review of the epidemiological literature. Allergy Asthma Immunol Res.

[12] Song WJ, Chang YS, Faruqi S, Kim JY, Kang MG, Kim S (2015). The global epidemiology of chronic cough in adults: a systematic review and meta-analysis. Eur Respir J.

[13] Irwin RS, Baumann MH, Bolser DC, Boulet LP, Braman SS, Brightling CE (2006). Diagnosis and management of cough executive summary: ACCP evidence-based clinical practice guidelines. Chest.

[14] Morice AH, Millqvist E, Belvisi MG, Bieksiene K, Birring SS, Chung KF (2014). Expert opinion on the cough hypersensitivity syndrome in respiratory medicine. Eur Respir J.

[15] Morice AH, Jakes AD, Faruqi S, Birring SS, McGarvey L, Canning B (2014). A worldwide survey of chronic cough: a manifestation of enhanced somatosensory response. Eur Respir J.

[16] Chung KF, McGarvey L, Mazzone SB (2013). Chronic cough as a neuropathic disorder. Lancet Respir Med.

[17] Irwin RS, French CL, Chang AB, Altman KW (2018). Classification of cough as a symptom in adults and management algorithms: CHEST guideline and expert panel report. Chest.

[18] Lai K, Pan J, Chen R, Liu B, Luo W, Zhong N (2013). Epidemiology of cough in relation to China. Cough (London, England).

[19] Lai K, Long L (2020). Current status and future directions of chronic cough in China. Lung.

[20] Long L, Lai K (2019). Characteristics of Chinese chronic cough patients. Pulm Pharmacol Ther.

[21] Li B, Lai K (2010). Epidemiology of chronic cough. Chin J Tuberc Respir Dis.

[22] Page MJ, McKenzie JE, Bossuyt PM, Boutron I, Hoffmann TC, Mulrow CD (2021). The PRISMA 2020 statement: an updated guideline for reporting systematic reviews. BMJ (Clin Res Ed).

[23] Hu J, Dong Y, Chen X, Liu Y, Ma D, Liu X (2015). Prevalence of suicide attempts among Chinese adolescents: a meta-analysis of cross-sectional studies. Compr Psychiatry.

[24] Rostom A, Dubé C, Cranney A, Saloojee N, Sy R, Garritty C (2004). Celiac disease. Evid Rep Technol Assess (Summ).

[25] Guyatt G, Oxman AD, Akl EA, Kunz R, Vist G, Brozek J (2011). GRADE guidelines: 1. Introduction-GRADE evidence profiles and summary of findings tables. J Clin Epidemiol.

[26] Jin Y. Comprehensive evaluation of the elderly Health in Xi’an: Fourth Military Medical University; 2013.

[27] Wang X, Deng FR, Lv HB, Wu SW, Guo XB (2011). Long-term effects of air pollution on the occurrence of respiratory symptoms in adults of Beijing. Beijing Da Xue Xue Bao Yi Xue Ban.

[28] Chen R, Lai K, Liu C-l, Luo W, Zhong N (2006). An epidemiologic study of cough in young college students in Guangzhou. Zhonghua liu xing bing xue za zhi = Zhonghua liuxingbingxue zazhi.

[29] Jin-Yu W, Sheng L, Shi-Gong W, Ke-Zheng S (2012). Effects of dust pollution on respiratory symptoms of long-term exposure population. J Lanzhou Univ Nat Sci.

[30] Lai CK, Ho SC, Lau J, Yuen YK, Ho SS, Chan CH (1995). Respiratory symptoms in elderly Chinese living in Hong Kong. Eur Respir J.

[31] Li JC, Zhang M, Li YC, Duan XL, Wang LM. Prevalence and influencing factors of respiratory symptoms among people aged 40 years and above in China. Zhonghua liu xing bing xue za zhi = Zhonghua liuxingbingxue zazhi. 2017. 10.3760/cma.j.issn.0254-6450.2018.06.018 29936748

[32] Liang-ping L, Wu C, Yang-chun X, Xiong-feng Y, Chun-feng R (2013). Epidemiological investigation of Shenzhen Pingshan New District 1468 workers with cough. Chin Manip Rehabil Med.

[33] Venners SA, Wang B, Ni J, Jin Y, Yang J, Fang Z (2001). Indoor air pollution and respiratory health in urban and rural China. Int J Occup Environ Health.

[34] Wen P, Ying W (2011). Cough status and etiology of 2588 college students. Chin J S chool Doctor.

[35] Zhang J, Qian Z, Kong L, Zhou L, Yan L, Chapman RS (1999). Effects of air pollution on respiratory health of adults in three Chinese cities. Arch Environ Health.

[36] Koo LC, Ho JH, Matsuki H, Shimizu H, Mori T, Tominaga S (1988). A comparison of the prevalence of respiratory illnesses among nonsmoking mothers and their children in Japan and Hong Kong. Am Rev Respir Dis.

[37] Wilson D, Takahashi K, Pan G, Chan CC, Zhang S, Feng Y (2008). Respiratory symptoms among residents of a heavy-industry province in China: prevalence and risk factors. Respir Med.

[38] Hu ZW, Zhao YN, Cheng Y, Guo CY, Wang X, Li N (2016). Living near a major road in Beijing: association with lower lung function, airway acidification, and chronic cough. Chin Med J (Engl).

[39] Zhang H, Dong L, Kang YK, Lu Y, Wei HH, Huang J (2018). Epidemiology of chronic airway disease: results from a cross-sectional survey in Beijing. China J Thorac Dis.

[40] Huang JH, Xu PS, Li JW, Li MX, Zhang TT, Luo X (2017). An epidemiological survey of chronic cough with no obvious abnormality in chest X-ray. Guangdong Med J..

[41] Huang DM, Xiao XX, Fu SM, Luo CM, Wang KM, Wang YH (2014). Incidence of wheezing and chronic cough in children aged 3–14 years in rural and urban areas of Zhongshan, China: a questionnaire survey. Zhongguo dang dai er ke za zhi = Chin J Contemp Pediatr.

[42] Salo PM, Xia J, Johnson CA, Li Y, Avol EL, Gong J (2004). Indoor allergens, asthma, and asthma-related symptoms among adolescents in Wuhan, China. Ann Epidemiol.

[43] Cai X, Luo C, Luo Y (2003). Epidemiological survey of the children with respiratory diseases. J Clin Pediatr..

[44] Fan M-Y, Tang X, Huang W, Dai H, Liu X-C, Xia Y-Y (2017). Effect of air pollution on respiratory health in school-aged children in the main urban area of Chongqing, China. Zhongguo dang dai er ke za zhi = Chin J Contemp Pediatr.

[45] Gao Y, Chan EYY, Li L, Lau PWC, Wong TW (2014). Chronic effects of ambient air pollution on respiratory morbidities among Chinese children: a cross-sectional study in Hong Kong. BMC Public Health.

[46] Guang-hui D. An epidemiologic study of affection of air pollution on respiratory health in children living in seven cities of Liaoning Province: China Medical University; 2004.

[47] Jin-gui W, Chun-jin N, Zu-jia Z, Li-ming W, Guo-liang L (2009). Study on environmental risk factors for chronic cough in school children in urban, Shanghai. Chin J Child Health Care.

[48] Kang-lu G, Hai-lin Z, Xiao-guang H, Chang-rong L (2012). A controlled study on the prevalence and risk factors of chronic cough in children. Zhejiang Prev Med.

[49] Liao MTW (2017). Epidemiological analysis of chronic cough in children in Area of Upper and Neighboring Dam in northern Hebei Province. Hainan Med J.

[50] Li Z, Dong-ming H, Shao-zhen Q, Xu-feng L, Si-mao F (2012). Epidemiology of chronic cough in children aged 2 to 12 in Zhongshan. Guangdong Med J..

[51] Liu Rong DG, Hou Shuwen, et al. Study of affection of outdoor air pollution on respiratory health among children. Chin J Public Health 2005;21(5).

[52] Niu C, Wu J, Zhuang ZZ (2010). Prevalence of respiratory symptoms and diseases among children and adolescent in urban Shanghai. Chin J Sch Health.

[53] Pan G, Zhang S, Feng Y, Takahashi K, Kagawa J, Yu L (2010). Air pollution and children's respiratory symptoms in six cities of Northern China. Respir Med.

[54] Xi Shu-hua SW, Ye L (2002). Analysis of the effect of air pollution on school children respiratory health. Mod Prev Med.

[55] Xi S, Sun W, Ye L (2003). Health status of children's respiratory systems and analysis of influencial factors in Benxi. J Environ Health..

[56] Zhang JJ, Hu W, Wei F, Wu G, Korn LR, Chapman RS (2002). Children's respiratory morbidity prevalence in relation to air pollution in four Chinese cities. Environ Health Perspect.

[57] Zhu YD, Wei JR, Huang L, Wang SH, Tian HM, Guo XB (2015). Comparison of respiratory diseases and symptoms among school-age children in areas with different levels of air pollution. Beijing Da Xue Xue Bao Yi Xue Ban.

[58] Li Sheng WJ, Wang Y (2014). Analysis on influencing factors of respiratory system diseases and symptoms of school children in Yuzhong county of Lanzhou. J Environ Health.

[59] Dong G, Ding H-l, Ma Y, Jin J, Cao Y, Zhao Y (2008). Housing characteristics, home environmental factors and respiratory health in 14,729 Chinese children. Revue d Epidemiologie et de Sante Publique..

[60] Wang D, Qian Z, Wang J, Yang M, Lee YL, Liu F (2014). Gender-specific differences in associations of overweight and obesity with asthma and asthma-related symptoms in 30 056 children: result from 25 districts of Northeastern China. J Asthma.

[61] China NBoSotPsRo (2011). Main data bulletin of the sixth national population census, 2010 (No. 1). Chin J Fam Plan.

[62] Maher SA, Birrell MA, Adcock JJ, Wortley MA, Dubuis ED, Bonvini SJ (2015). Prostaglandin D2 and the role of the DP1, DP2 and TP receptors in the control of airway reflex events. Eur Respir J.

[63] Deng Z, Zhou W, Sun J, Li C, Zhong B, Lai K (2018). IFN-γ enhances the cough reflex sensitivity via calcium influx in vagal sensory neurons. Am J Respir Crit Care Med.

[64] Mazzone SB, Undem BJ (2016). Vagal afferent innervation of the airways in health and disease. Physiol Rev.

[65] Chen Z, Sun L, Chen H, Gu D, Zhang W, Yang Z (2018). Dorsal vagal complex modulates neurogenic airway inflammation in a guinea pig model with esophageal perfusion of HCl. Front Physiol.

[66] Mazzone SB, Farrell MJ (2019). Heterogeneity of cough neurobiology: clinical implications. Pulm Pharmacol Ther.

[67] Driessen AK, McGovern AE, Behrens R, Moe AAK, Farrell MJ, Mazzone SB (2020). A role for neurokinin 1 receptor expressing neurons in the paratrigeminal nucleus in bradykinin-evoked cough in guinea-pigs. J Physiol.

[68] Khalid S, Murdoch R, Newlands A, Smart K, Kelsall A, Holt K (2014). Transient receptor potential vanilloid 1 (TRPV1) antagonism in patients with refractory chronic cough: a double-blind randomized controlled trial. J Allergy Clin Immunol.

[69] Belvisi MG, Birrell MA, Wortley MA, Maher SA, Satia I, Badri H (2017). XEN-D0501, a novel transient receptor potential vanilloid 1 antagonist, does not reduce cough in patients with refractory cough. Am J Respir Crit Care Med.

[70] Smith JA, McGarvey LPA, Badri H, Satia I, Warren F, Siederer S (2017). Effects of a novel sodium channel blocker, GSK2339345, in patients with refractory chronic cough. Int J Clin Pharmacol Ther.

[71] Smith J, Allman D, Badri H, Miller R, Morris J, Satia I (2020). The Neurokinin-1 receptor antagonist orvepitant is a novel antitussive therapy for chronic refractory cough: results from a Phase 2 Pilot Study (VOLCANO-1). Chest.

[72] Smith JA, Kitt MM, Butera P, Smith SA, Li Y, Xu ZJ (2020). Gefapixant in two randomised dose-escalation studies in chronic cough. Eur Respir J.

[73] Diseases CSoR (2005). Guidelines for the diagnosis and treatment of cough (2005). Chin J Tuberc Respir Dis.

[74] Lai K (2009). Guidelines for the diagnosis and treatment of cough. Chin J Tuberc Respir Dis.

[75] Diseases CSoR. Guidelines for the diagnosis and treatment of cough (2015). Chin J Tuberc Respir Dis. 2015.

[76] Zang X, Lu Y, Yao H, Li FD, Zhang SC (2015). The temporal and spatial distribution characteristics of main air pollutants in China. Ecol Environ Sci.

[77] Kun X. The spatial analysis of air pollution in China: Huazhong University of Science & Technology; 2016.

[78] Weinberger M, Hurvitz M (2020). Diagnosis and management of chronic cough: similarities and differences between children and adults. F1000Research.

[79] McGarvey L, Gibson PG (2019). What is chronic cough? Terminology. J Allergy Clin Immunol Pract.

[80] Matsuba K, Thurlbeck WM (1972). A morphometric study of bronchial and bronchiolar walls in children. Am Rev Respir Dis.

[81] Canning BJ, Chang AB, Bolser DC, Smith JA, Mazzone SB, McGarvey L (2014). Anatomy and neurophysiology of cough: CHEST Guideline and Expert Panel report. Chest.

[82] Kantar A, Seminara M (2019). Why chronic cough in children is different. Pulm Pharmacol Ther.

[83] Diseases CSoR (2016). Guidelines for the diagnosis and treatment of cough 2015. Chin J Tuberc Respir Dis.

[84] Dicpinigaitis PV, Canning BJ (2020). Is there (will there be) a post-COVID-19 chronic cough?. Lung.

