# Supplementary material for: Prevalence of chronic cough in China: a systematic review and meta-analysis
Source: BMC Pulm Med. 2022 Feb 12;22:62. doi: 10.1186/s12890-022-01847-w (PMC8840780; doi:10.1186/s12890-022-01847-w)
Supplement: Supplementary file 1 — Additional file 1. Search strategies. [file 12890_2022_1847_MOESM1_ESM.pdf]

PubMed

#1 chronic cough[Title/Abstract]

#2 prolonged cough[Title/Abstract]

#3 persistent cough[Title/Abstract]

#4 refractory cough[Title/Abstract]

#5 chronic idiopathic cough[Title/Abstract]

#6 unexplained cough[Title/Abstract]

#7 "Cough"[Mesh]

#8 #1 OR #2 OR #3 OR #4 OR #5 OR #6 OR #7

#9 incidence[Title/Abstract]

#10 demograph*[Title/Abstract]

#11 etiolog*[Title/Abstract]

#12 epidemiolog*[Title/Abstract]

#13 prevalen*[Title/Abstract]

#14 #9 OR #10 OR #11 OR #12 OR #13

#15 China[All fields]

#16 #8 AND #14 AND #15

Web of Science

#1 TI=(chronic cough OR prolonged cough OR persistent cough OR refractory cough OR unexplained cough)

#2 AB=(chronic cough OR prolonged cough OR persistent cough OR refractory cough OR unexplained cough)

#3 #1 OR #2

#4 AB=(incidence OR demograph* OR epidemiolog* OR prevalen*)

#5 TI=(incidence OR demograph* OR epidemiolog* OR prevalen*)

#6 #4 OR #5

#7 TS=(China)

#8 #3 AND #6 AND #7

Cochrane Library

#1 (chronic cough):ti,ab,kw OR (prolonged cough):ti,ab,kw OR (persistent cough):ti,ab,kw OR (refractory cough):ti,ab,kw OR (unexplained cough):ti,ab,kw

#2 (incidence):ti,ab,kw OR (demograph*):ti,ab,kw OR (epidemiolog*):ti,ab,kw OR (prevalen*):ti,ab,kw

#3 (China)

#4 #1 AND #2 AND #3

Chinese biomedical literature service system

#1 "慢性咳嗽"[Commonly used field: intelligence] OR "长期咳嗽"[Commonly used field: intelligence] OR "持续咳嗽"[Commonly used field: intelligence] OR "难治性咳嗽"[Commonly used field: intelligence]

#2 "患病率"[[Commonly used field: intelligence] OR "流行"[Commonly used field: intelligence] OR "现状"[Commonly used field: intelligence]

#3 #1 AND #2

China National Knowledge Infrastructure

(TKA="慢性咳嗽" OR TKA="长期咳嗽" OR TKA="持续咳嗽" OR TKA="难治性咳嗽" OR TKA="慢性特发性咳嗽" OR TKA="咳嗽高敏感综合征") AND (TKA="患病率" OR TKA="流行" OR TKA="现状")

Wanfang Database

#1 主题:(慢性咳嗽) or 主题:(长期咳嗽) or 主题:(持续咳嗽) or 主题:(难治性咳嗽)

#2 主题:(患病率) or 主题:(流行) or 主题:(现状)

#3 #1 AND #2

VIP database

(M=慢性咳嗽 OR M=长期咳嗽 OR M=持续咳嗽 OR M=难治性咳嗽) AND (U=患病率 OR U=流行 OR U=现状 OR U=调查)
