# Supplementary material for: Prevalence of chronic cough in China: a systematic review and meta-analysis
Source: BMC Pulm Med. 2022 Feb 12;22:62. doi: 10.1186/s12890-022-01847-w (PMC8840780; doi:10.1186/s12890-022-01847-w)
Supplement: Supplementary file 2 — Additional file 2. Cross-sectional/prevalence study quality. [file 12890_2022_1847_MOESM2_ESM.docx]

**Additional file 2 Cross-Sectional/Prevalence Study Quality**

| **Item** | **Yes** | **No** | **Unclear** |
| --- | --- | --- | --- |
| 1) Define the source of information (survey, record review) |  |  |  |
| 2) List inclusion and exclusion criteria for exposed and unexposed subjects (cases and controls) or refer to previous publications |  |  |  |
| 3) Indicate time period used for identifying patients |  |  |  |
| 4) Indicate whether or not subjects were consecutive if not population-based |  |  |  |
| 5) Indicate if evaluators of subjective components of study were masked to other aspects of the status of the participants |  |  |  |
| 6) Describe any assessments undertaken for quality assurance purposes (e.g., te st/retest of primary outcome measurements) |  |  |  |
| 7) Explain any patient exclusions from analysis |  |  |  |
| 8) Describe how confounding was assessed and/or controlled. |  |  |  |
| 9) If applicable, explain how missing data were handled in the analysis |  |  |  |
| 10) Summarize patient response rates and completeness of data collection |  |  |  |
| 11) Clarify what follow-up, if any, was expected and the percentage of patients for which incomplete data or follow-up was obtained |  |  |  |
