# Supplementary material for: Prevalence of chronic cough in China: a systematic review and meta-analysis
Source: BMC Pulm Med. 2022 Feb 12;22:62. doi: 10.1186/s12890-022-01847-w (PMC8840780; doi:10.1186/s12890-022-01847-w)
Supplement: Supplementary file 3 — Additional file 3. Exclusion with reasons. [file 12890_2022_1847_MOESM3_ESM.docx]

**Additional file 3 Exclusion with reasons**

| Title | Published year | Author | Reason | Notes |
| --- | --- | --- | --- | --- |
| [A Study of the risk Factors and impact of air pollution on Respiratory Health of Adults in 6 Cities of Liaoning Province] | 2006 | Liu J | The time, spot and population of the subjects in Wilson et al 2008 and Liu J 2006 were same, so these two articles were duplicate. Liu J 2006 is a dissertation published in Chinese while Wilson et al 2008 was published in an English journal. Considering that paper published in English would be more accessible to researchers from other country, we replaced Liu J 2006 with Wilson et al 2008. | - |
| Occupational and environmental risk factors for respiratory symptoms in rural Beijing, China | 2002 | L-X. Zhang | The purpose of this article is to study the risk factors of chronic cough. The prevalence of chronic cough is not reported in the main text, but is reported in a table with the prevalence of 1.9 (1.8-2.0). However, the sample size in the table is 22561, while the sample size presented in results section is 22528. Therefore, we think that the prevalence presented in this paper is unreliable. In addition, the article includes residents aged ≥ 15 years old, but the diagnostic criteria of chronic cough are based on the criteria of adults. All things considered, we exclude this article. | - |
| Effects of environmental tobacco smoke on respiratory health of boys and girls from kindergarten: results from 15 districts of northern China | 2007 | Dong, G. H. | The study population of Dong et al 2008 was overlapped with the study population of Dong et al 2007. Dong et al 2007 was conducted in April of 2007 in four cities of Liaoning Province (Shenyang, Dalian, Benxi and Anshan). The age of the population was 1 to 6 years old and the sample size was 6053. | Dong et al 2008 was conducted in April of 2007. Shenyang, Dalian and Anshan were randomly selected from Liaoning Province. A total of 14729 children, of which 3945 preschool children (1-6 years old) and 10784 primary school students (7-13 years old), were investigated. Since Dong’s research group published 6 articles based on the survey conducted in the same place in April 2007, we only included the study with the largest range of age and sample size. |
| Pets keeping in home, parental atopy, asthma, and asthma-related symptoms in 12,910 elementary school children from northeast China | 2009 | G.-H. Dong | The study population of Dong et al 2008 was overlapped with the study population of Dong et al 2009. Dong et al 2009 was conducted in April of 2007 in three cities of Liaoning Province (Shenyang, Dalian and Anshan). The age of the population was 7 to 13 years old and the sample size was 12910. |  |
| Effects of Housing Characteristics and Home Environmental Factors on Respiratory Symptoms of 10,784 Elementary School Children from  Northeast China | 2008 | G.-H. Dong | The study population of Dong et al 2008 was overlapped with the study population of this study. This study was conducted in April of 2007 in three cities of Liaoning Province (Shenyang, Dalian and Anshan). The age of the population was 6 to 13 years old and the sample size was 10784. |  |
| Housing characteristics, home environmental factors and respiratory health in 3945 preschool children in China | 2008 | Guang-Hui Dong | The study population of Dong et al 2008 was overlapped with the study population of this study. This study was conducted in April of 2007 in three cities of Liaoning Province (Shenyang, Dalian and Anshan). The age of the population was 1 to 6 years old and the sample size was 3945. |  |
| [Interactive effects of environmental tobacco smoke and pets ownership on respiratory diseases and symptoms in children] | 2013 | ZHAO Y | The study population of Dong et al 2008 was overlapped with the study population of this study. The corresponding author of this study is Dong. This study was conducted in April of 2007 in Shenyang city of Liaoning Province. The age of the population was 8.08±2.88 years old and the sample size was 8733. |  |
| [Interaction effects of breastfeeding and passive smoking on asthma and asthma-related symptoms among children] | 2013 | Liu Yu-qin | The study population of Wang et al 2014 was overlapped with the study population of this study. The corresponding author of the two studies are Dong. This study was conducted in 25 primary schools and 50 kindergartens in seven cities of Liaoning Province in 2009. The age of the population was 2 to 14 years old and the sample size was 31049. | The corresponding author of study “Da Wang” is Dong. The survey was carried out in seven cities randomly selected from Liaoning Province (Shenyang, Dalian, Anshan, Fushun, Benxi, Liaoyang and Yingkou) in April 2009. A total of 31049 subjective from 50 kindergartens and 25 primary schools were included. The range of age was 2 to 14 years old. After excluding the subjects resided in the above seven cities less than 2 years or with insufficient data, 30056 subjects were included in final analysis. Considering the largest range of age and largest sample size of this study, we include Wang et al 2014 and exclude other studies with data overlapped with Wang et al 2014. Although Liu et al 2013 published in Chinese is similar to Wang et al 2014 in sample size and prevalence, we only include Wang et al 2014 in order to facilitate researchers from other countries to read the original text. |
| Asthma and asthma related symptoms in 23,326 Chinese children in relation to indoor and outdoor environmental factors: The Seven Northeastern Cities (SNEC) Study | 2014 | Fan Liu | The study population of Da Wang 2014 was overlapped with the study population of this study. The corresponding author of the studies is Guang Hui Dong. This study was conducted in 25 primary schools in seven cities of Liaoning Province in April 2009. The age of the population was 6 to 13 years old and the sample size was 23326. |  |
| Effects of Outdoor and Indoor Air Pollution on Respiratory Health of Chinese Children from 50 Kindergartens | 2013 | Miao-Miao Liu | The study population of Wang et al 2014 was overlapped with the study population of this study. The corresponding author of the studies is Dong. This study was conducted in 50 kindergartens in seven cities of Liaoning Province in April 2009. The age of the population was 3 to 7 years old and the sample size was 6730. |  |
| Exposure to Secondhand Tobacco Smoke Enhances Respiratory Symptoms and Responses to Animals in 8,819 Children in Kindergarten:  Results from 25 Districts in Northeast China | 2011 | Guang-Hui Dong | The study population of Wang et al 2014 was overlapped with the study population of this study. This study was conducted in 50 kindergartens in seven cities of Liaoning Province in April 2009. The age of the population was 2 to 6 years old and the sample size was 8819. |  |
| Gender Differences and Effect of Air Pollution on Asthma in Children with and without Allergic Predisposition: Northeast Chinese Children Health Study | 2011 | Guang-Hui Dong | The study population of Wang et al 2014 was overlapped with the study population of this study. This study was conducted in seven cities of Liaoning Province in April 2009. The age of the population was 3 to 12 years old and the sample size was 30139. |  |
