# Supplementary material for: Prevalence of chronic cough in China: a systematic review and meta-analysis
Source: BMC Pulm Med. 2022 Feb 12;22:62. doi: 10.1186/s12890-022-01847-w (PMC8840780; doi:10.1186/s12890-022-01847-w)
Supplement: Supplementary file 5 — Additional file 5. Methodology of studies included in the meta-analysis. [file 12890_2022_1847_MOESM5_ESM.docx]

**Additional file 5 Methodology of studies included in the meta-analysis**

| **Study** | **Survey method** | **Inclusion and exclusion criteria** | **Define** | **Time period used for identifying patients** | **Subjects were consecutive** | **Quality assurance/retest** | **Confounding was assessed and/or controlled** | **Period prevalence/point prevalence** | **Sample source** |
| --- | --- | --- | --- | --- | --- | --- | --- | --- | --- |
| LINDA C. KOO 1988 | questionnaire | Not mentioned | On the basis of longevity | 10 days | consecutive | Not mentioned | Not mentioned | point prevalence | elementary schools |
| C.K.W. Lai 1995 | questionnaire | Not mentioned | Not mentioned | 1 year | inconsecutive | Not mentioned | Not mentioned | point prevalence | community-based |
| ZHANG JF 1999 | questionnaire | Not mentioned | On the basis of longevity | 3 months | consecutive | Not mentioned | Not mentioned | point prevalence | elementary schools |
| Venners 2001 | questionnaire | Not mentioned | On the basis of longevity | 3 months | consecutive | Not mentioned | Not mentioned | period prevalence | community-based |
| ZHANG JF 2002 | questionnaire | Not mentioned | On the basis of longevity | 3 years | consecutive | Not mentioned | Not mentioned | point prevalence | elementary schools |
| XI SH 2002 | questionnaire | resided in the relevant monitoring district for at least 3 years | On the basis of longevity | half a month | inconsecutive | Not mentioned | Not mentioned | point prevalence | elementary schools |
| CAI XH 2003 | questionnaire | Not mentioned | On the basis of guidelines | 1 year | consecutive | Not mentioned | Not mentioned | point prevalence | community-based |
| XI SH 2003 | questionnaire | Not mentioned | On the basis of longevity | half a month | inconsecutive | Not mentioned | Not mentioned | point prevalence | elementary schools |
| DONG GH 2004 | questionnaire | school-age children who resided in the relevant monitoring district for at least 3 years, and young children at least 1 years | On the basis of longevity | Not mentioned | consecutive | Not mentioned | Investigator training | period prevalence | kindergarten/elementary schools |
| Salo MS 2004 | questionnaire | Not mentioned | On the basis of longevity | 3 months | consecutive | Not mentioned | Not mentioned | period prevalence | secondary school |
| LIU R 2005 | questionnaire | school-age children who resided in the relevant monitoring district for at least 3 years, and young children at least 1 years | Not mentioned | Not mentioned | consecutive | Not mentioned | Not mentioned | point prevalence | kindergarten/elementary schools |
| CHEN RC 2006 | questionnaire interview | Not mentioned | On the basis of longevity | 2 weeks | consecutive | Not mentioned | Investigator training | point prevalence | university |
| Donald Wilson 2008 | questionnaire | schools that were located within 1 km of three environmental monitoring station | On the basis of longevity | 6 months | consecutive | Not mentioned | Not mentioned | point prevalence | kindergarten/elementary schools |
| Guang-Hui Dong 2008 | questionnaire | Not mentioned | On the basis of longevity | 1 month | consecutive | approved by the Human Ethics Committee, informed consent | Not mentioned | period prevalence | kindergarten/elementary schools |
| WU JG 2009 | questionnaire | resided in the relevant monitoring district for at least 2 years | On the basis of longevity | Not mentioned | consecutive | Not mentioned | Not mentioned | period prevalence | kindergarten/elementary schools/secondary school |
| Guowei Pan 2010 | questionnaire | Not mentioned | On the basis of longevity | 6 months | consecutive | Not mentioned | Not mentioned | period prevalence | kindergarten/elementary schools |
| NIU CJ 2010 | questionnaire | Not mentioned | On the basis of longevity | Not mentioned | consecutive | logical error correction, double entry and validation | Investigator training | point prevalence | kindergarten/elementary schools/secondary school |
| PAN W 2011 | questionnaire | Not mentioned | On the basis of longevity | Not mentioned | consecutive | Not mentioned | Not mentioned | point prevalence | university |
| WANG X 2011 | questionnaire | resided in the relevant monitoring district for at least 2 years | Not mentioned | 1 month | consecutive | logical error correction, double entry and validation | Investigator training | point prevalence | elementary schools |
| GAO KL 2012 | questionnaire | resided in the relevant monitoring district for at least half a year | On the basis of longevity | Not mentioned | consecutive | Not mentioned | Investigator training | point prevalence | kindergarten/elementary schools/secondary school/community |
| WANG JY 2012 | questionnaire | without a dust-exposure history | On the basis of longevity | 1 month | inconsecutive | logical error correction, double entry and validation | Not mentioned | period prevalence | community-based |
| ZHANG L 2012 | questionnaire | resided in the relevant monitoring district for at least 2 years | On the basis of longevity | 3 years | consecutive | Not mentioned | Not mentioned | period prevalence | kindergarten/elementary schools |
| LI LP 2013 | questionnaire interview | Not mentioned | On the basis of longevity | 2 weeks | consecutive | The survey schedule was adjusted and modified after the preliminary test | Not mentioned | point prevalence | examination center |
| YUE J 2013 | questionnaire interview | healthy elderly populations who without severe cognitive impairment, severe cardiac and pulmonary insufficiency or organ failure | Not mentioned | 16 months | inconsecutive | quality audit and follow up again the next day if necessary | Investigator training | point prevalence | community/hospitalization for physical examination |
| HUANG DM 2014 | questionnaire | resided in the relevant monitoring district for at least half a year | On the basis of longevity | Not mentioned | population-based | quality audit, and 5% data is checked by verification | Investigator training | point prevalence | elementary schools/secondary school |
| Da Wang 2014 | questionnaire | Not mentioned | On the basis of longevity | 1 month | consecutive | approved by the Human Ethics Committee, informed consent | Not mentioned | period prevalence | kindergarten/elementary schools |
| LI S 2014 | questionnaire | resided in the relevant monitoring district for at least 3 years | On the basis of longevity | 2 months | consecutive | quality audit, telephone return visit, logical error correction, double entry and validation | Investigator training | period prevalence | elementary schools |
| Yang Gao 2014 | questionnaire | had been currently living in the district where their school was located for more than 12 consecutive months prior to the study | On the basis of longevity | 3 months | consecutive | Not mentioned | Exclusion of confounding factors | point prevalence | elementary schools |
| ZHU YD 2015 | questionnaire | Not mentioned | Not mentioned | Not mentioned | consecutive | logical error correction, double entry and validation | Investigator training | point prevalence | elementary schools |
| FAN MY 2017 | questionnaire | Individuals with additional congenital malformations and defects, congenital cardiopulmonary diseases, asthma, and children with respiratory symptoms at the time of the measurement were excluded. | Not mentioned | Not mentioned | inconsecutive | quality audit, telephone return visit, logical error correction | Investigator training | point prevalence | elementary schools |
| HUANG JH 2017 | questionnaire interview | Not mentioned | On the basis of guidelines | 4 months | population-based | Not mentioned | Investigator training | point prevalence | community-based |
| LI M 2017 | questionnaire | resided in the relevant monitoring district for at least 2 years | On the basis of longevity | 1 year | inconsecutive | Not mentioned | Not mentioned | period prevalence | community-based |
| LI JC 2018 | questionnaire | Not mentioned | On the basis of longevity | 6 months | consecutive | quality audit, logical error correction | Investigator training | period prevalence | community-based |
| Zhan‑Wei Hu 2016 | questionnaire | resided at least 3 years | On the basis of longevity | 3 years | population-based | Not mentioned | Not mentioned | period prevalence | community-based |
| Hong Zhang 2018 | questionnaire | Not mentioned | On the basis of longevity | 9 months | population-based | sample size estimate, approved by the Human Ethics Committee, informed consent, double entry and validation | Investigator training | point prevalence | community-based |
