# Supplementary material for: Prevalence of chronic cough in China: a systematic review and meta-analysis
Source: BMC Pulm Med. 2022 Feb 12;22:62. doi: 10.1186/s12890-022-01847-w (PMC8840780; doi:10.1186/s12890-022-01847-w)

**Additional file 6**

**Figure S1** Distribution of children with chronic cough across Mainland China. NOTE: Red star in the map represents Beijing City. The map was developed in XL Toolbox NG by ourselves, without the conflict of copyright.


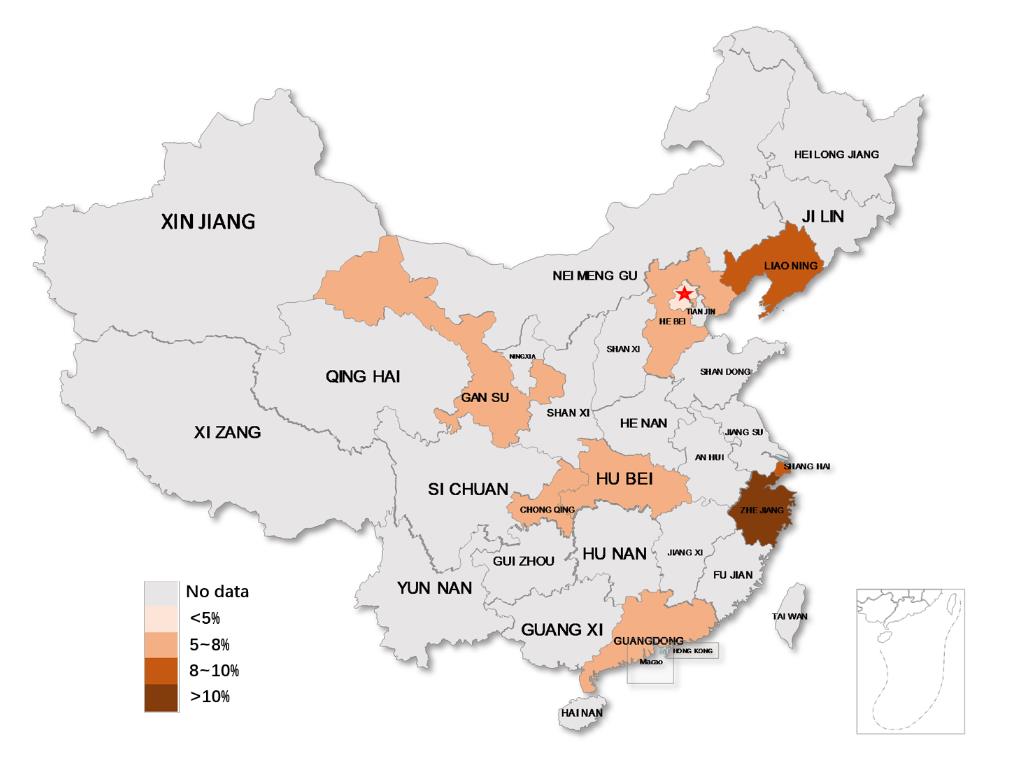


**Figure S2** Pooled chronic cough prevalence of adults stratified by region. Abbreviations: CI, confidence intervals. NOTE: The three author labels of ZHANG JF 1999 are from the same literature, and the two author labels of Venners 2001 are from the same literature.


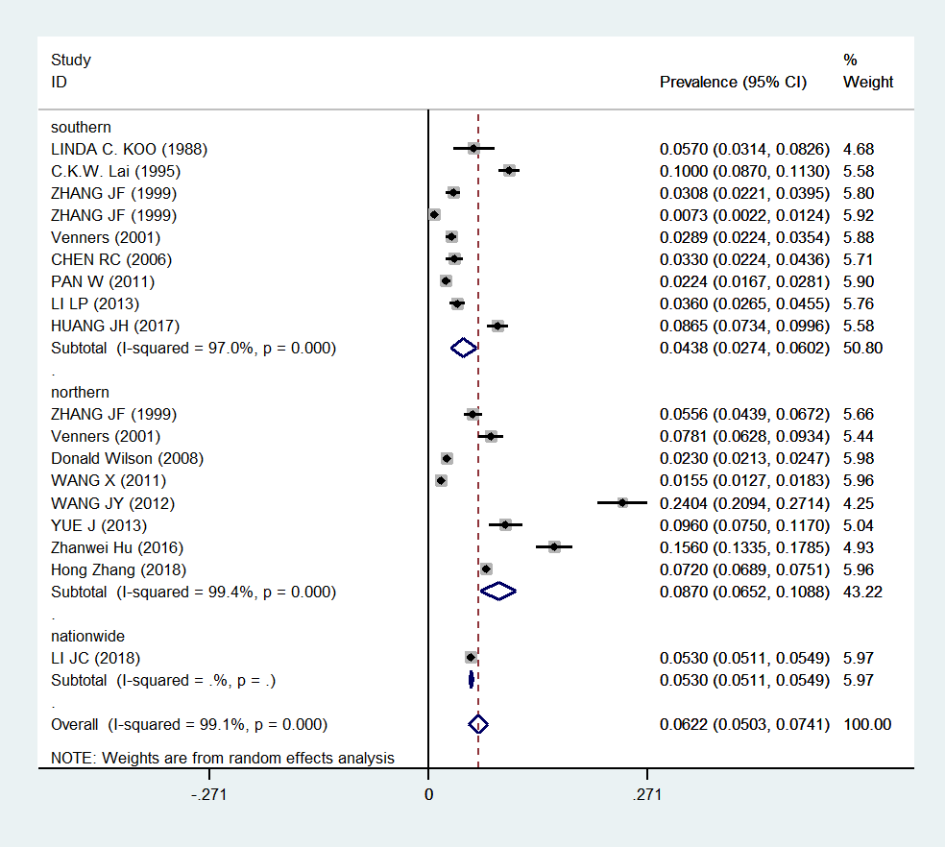


**Figure S3** Pooled chronic cough prevalence of adults stratified by diagnostic criteria. Abbreviations: CI, confidence intervals. NOTE: The three author labels of ZHANG JF 1999 are from the same literature, and the two author labels of Venners 2001 are from the same literature.


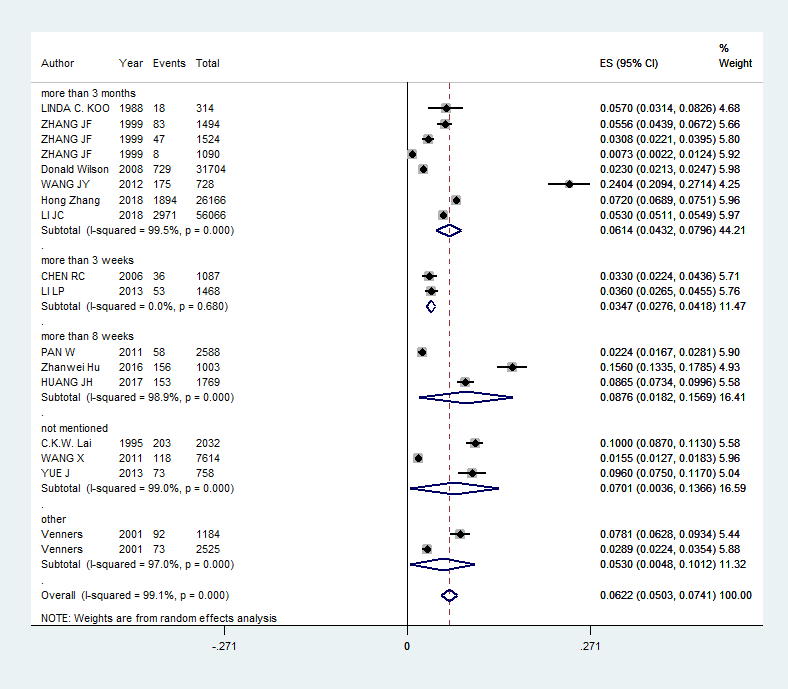


**Figure S4** Pooled chronic cough prevalence of adults stratified by year of publication. Abbreviations: CI, confidence intervals. NOTE: The three author labels of ZHANG JF 1999 are from the same literature, and the two author labels of Venners 2001 are from the same literature.


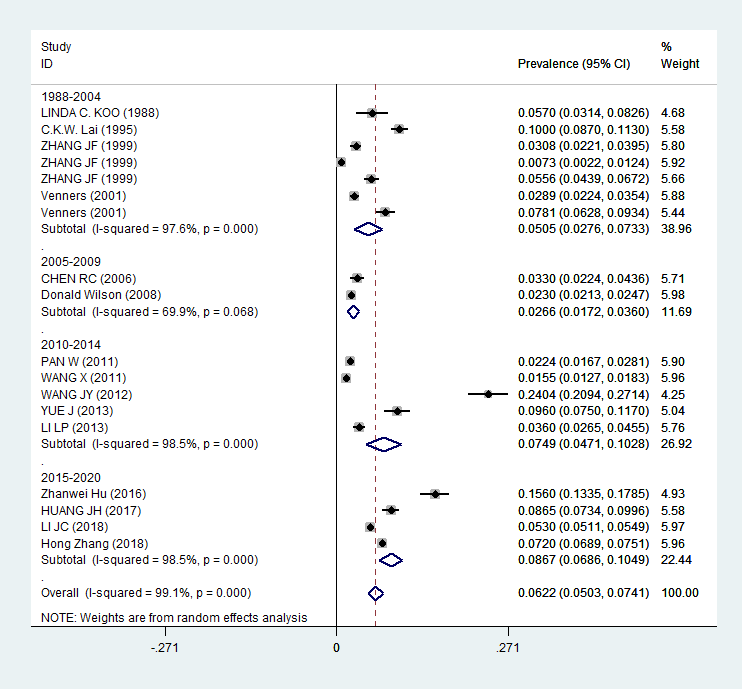


**Figure S5** Pooled chronic cough prevalence of adults stratified by age. Abbreviations: CI, confidence intervals. NOTE: The three author labels of ZHANG JF 1999 are from the same literature, and the two author labels of Venners 2001 are from the same literature.


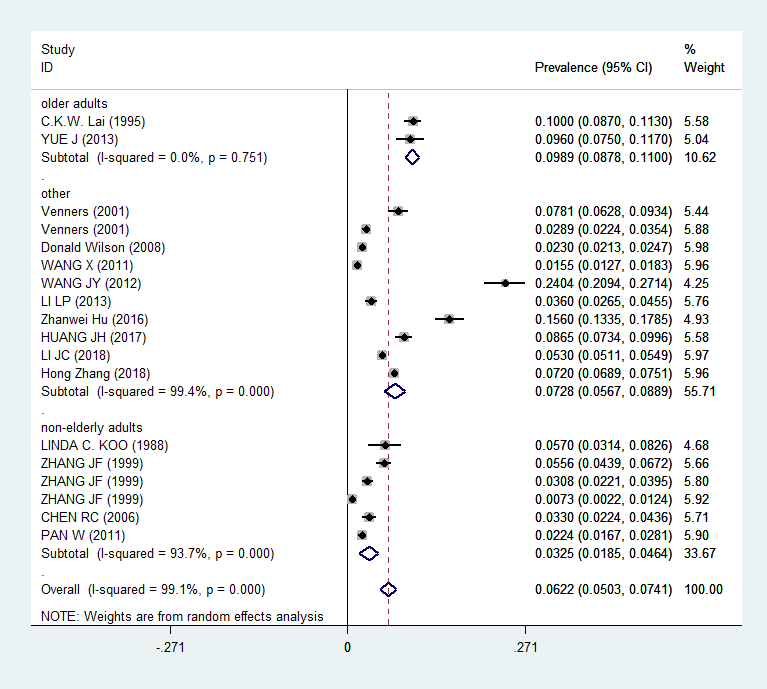


**Figure S6** Pooled chronic cough prevalence of adults stratified by sampling methods. Abbreviations: CI, confidence intervals. NOTE: The three author labels of ZHANG JF 1999 are from the same literature, and the two author labels of Venners 2001 are from the same literature.


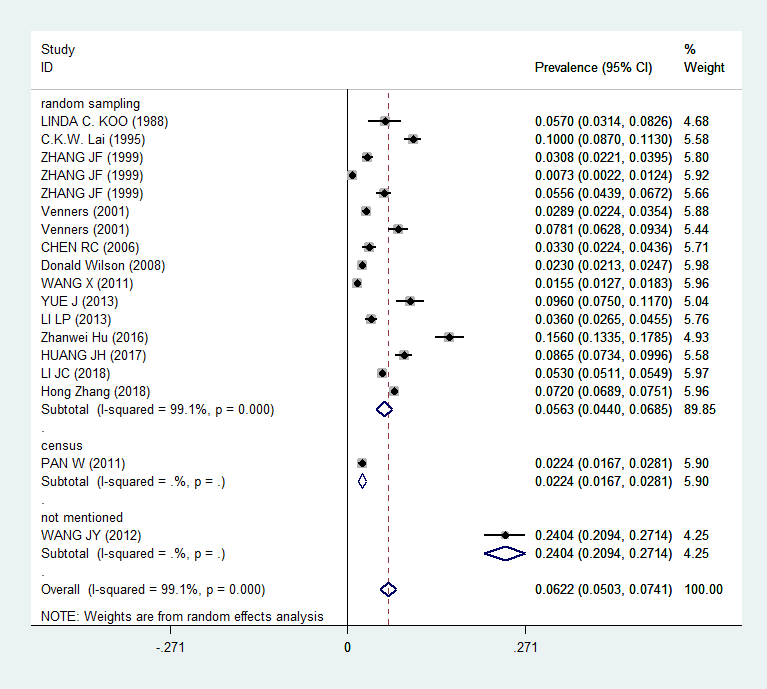


**Figure S7** Pooled chronic cough prevalence of adults stratified by sample size. Abbreviations: CI, confidence intervals; ES, Effect Size. NOTE: The three author labels of ZHANG JF 1999 are from the same literature, and the two author labels of Venners 2001 are from the same literature.


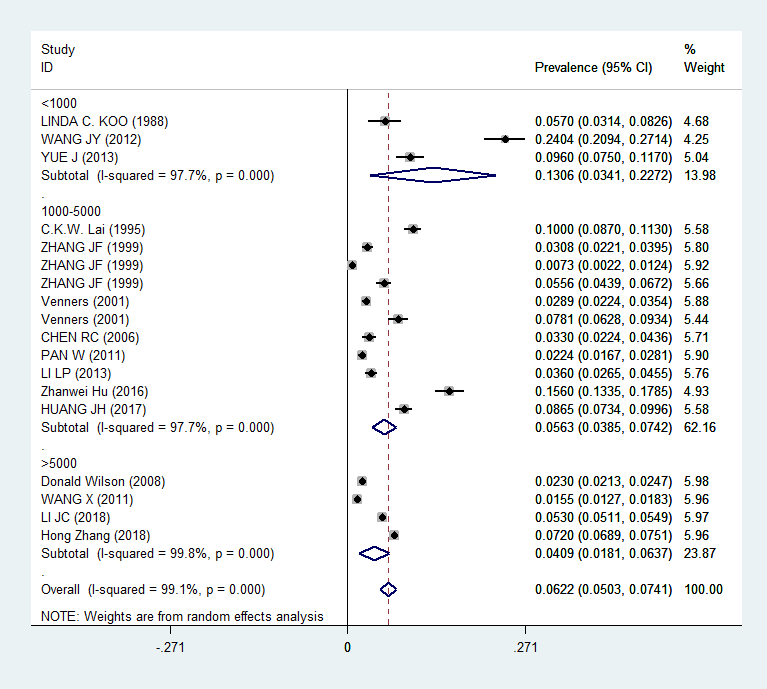


**Figure S8** Pooled chronic cough prevalence of adults stratified by prevalence definitions. Abbreviations: CI, confidence intervals; ES, Effect Size. NOTE: The three author labels of ZHANG JF 1999 are from the same literature, and the two author labels of Venners 2001 are from the same literature.


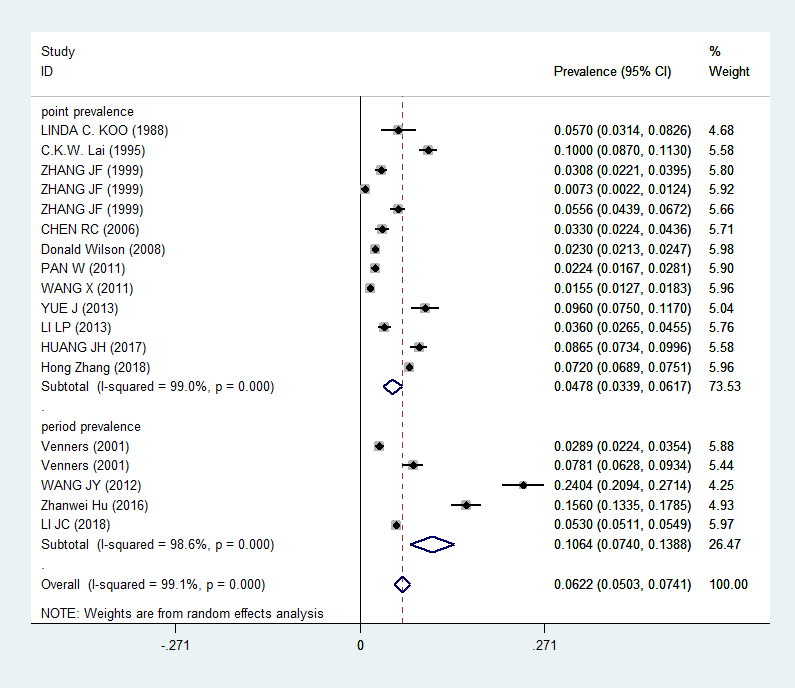


**Figure S9** Pooled chronic cough prevalence of adults stratified by chronic cough definitions. Abbreviations: CI, confidence intervals; ES, Effect Size. NOTE: The three author labels of ZHANG JF 1999 are from the same literature, and the two author labels of Venners 2001 are from the same literature.


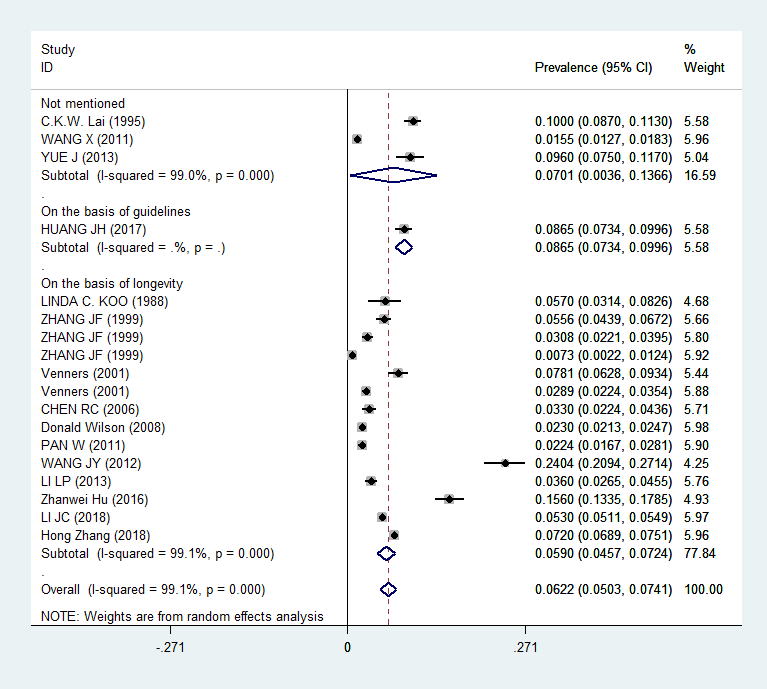


**Figure S10** Pooled chronic cough prevalence of adults stratified by quality of articles assessed by AHRQ. Abbreviations: CI, confidence intervals; ES, Effect Size. NOTE: The three author labels of ZHANG JF 1999 are from the same literature, and the two author labels of Venners 2001 are from the same literature.


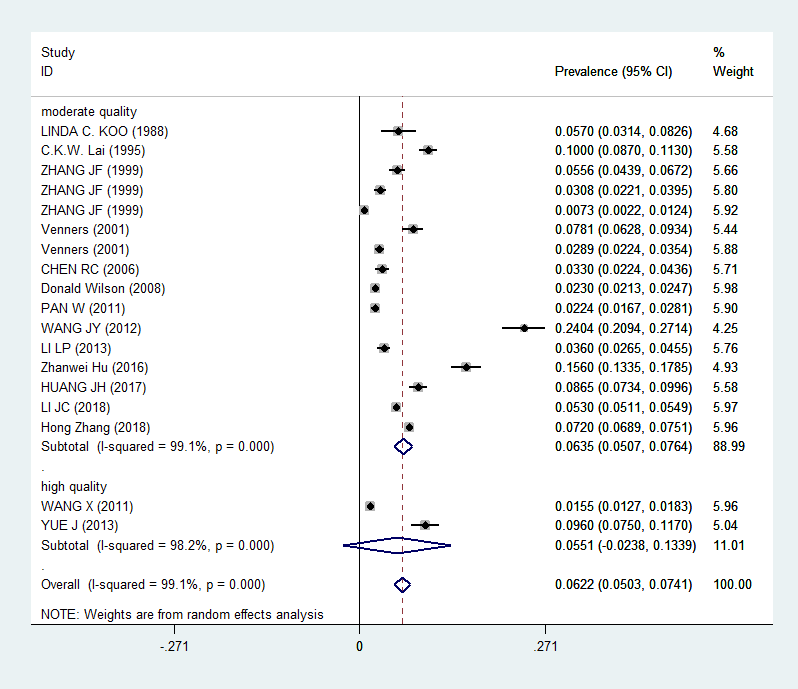


**Figure S11** Pooled chronic cough prevalence of children stratified by region. Abbreviations: CI, confidence intervals. NOTE: The four author labels of ZHANG JF 2002 are from the same literature.


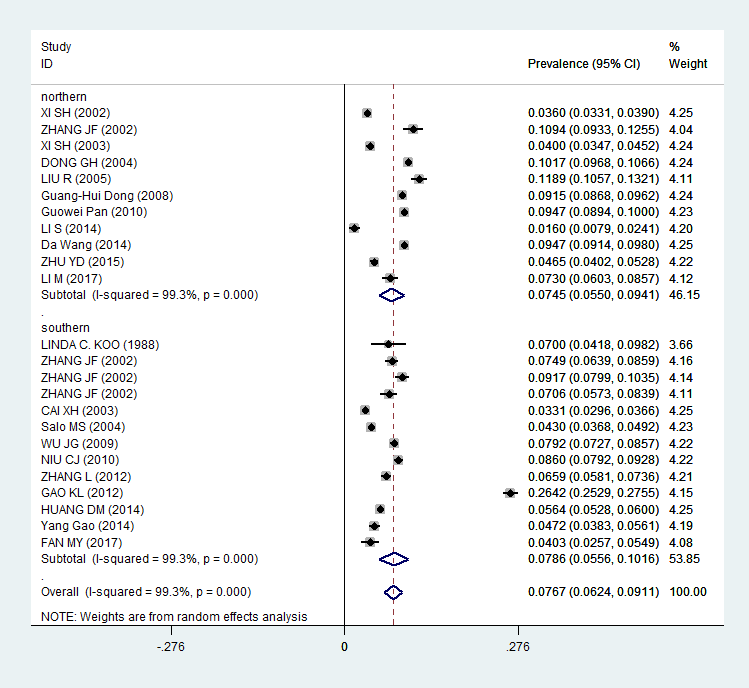


**Figure S12** Pooled chronic cough prevalence of children stratified by diagnostic criteria. Abbreviations: CI, confidence intervals. NOTE: The four author labels of ZHANG JF 2002 are from the same literature.


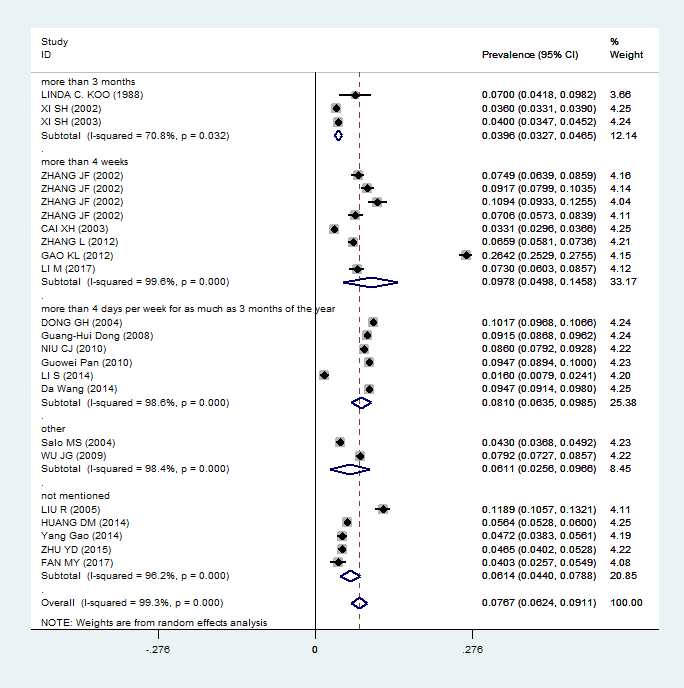


**Figure S13** Pooled chronic cough prevalence of children stratified by year of publication. Abbreviations: CI, confidence intervals. NOTE: The four author labels of ZHANG JF 2002 are from the same literature.


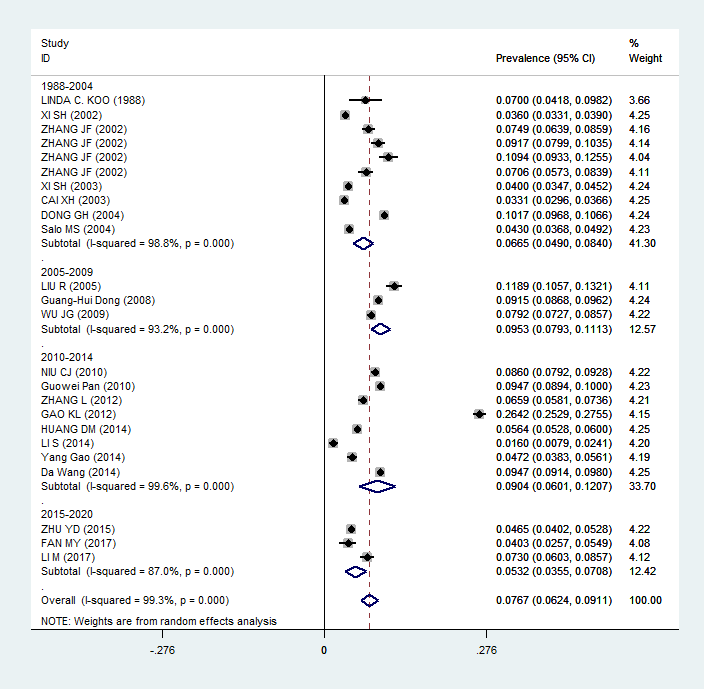


**Figure S14** Pooled chronic cough prevalence of children stratified by sample size. Abbreviations: CI, confidence intervals. NOTE: The four author labels of ZHANG JF 2002 are from the same literature.


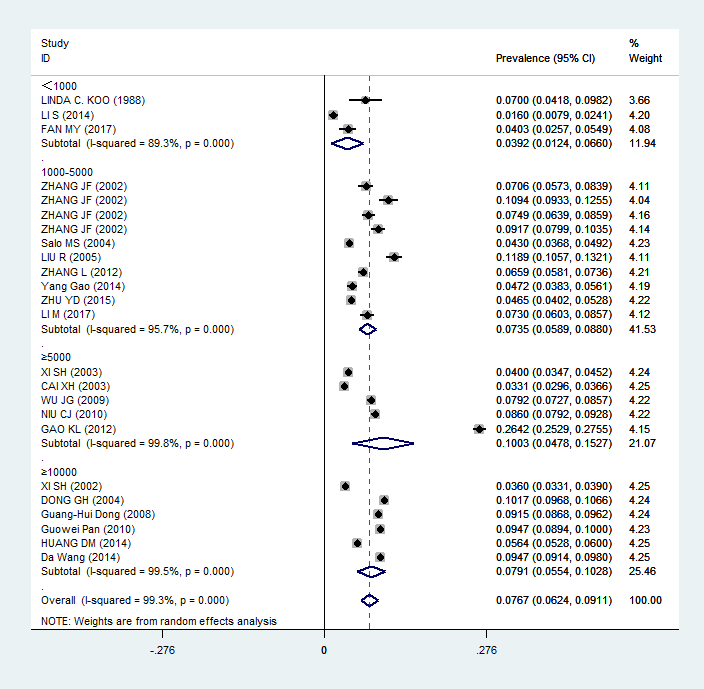


**Figure S15** Pooled chronic cough prevalence of children stratified by chronic cough definitions. Abbreviations: CI, confidence intervals; ES, Effect Size. NOTE: The four author labels of ZHANG JF 2002 are from the same literature.


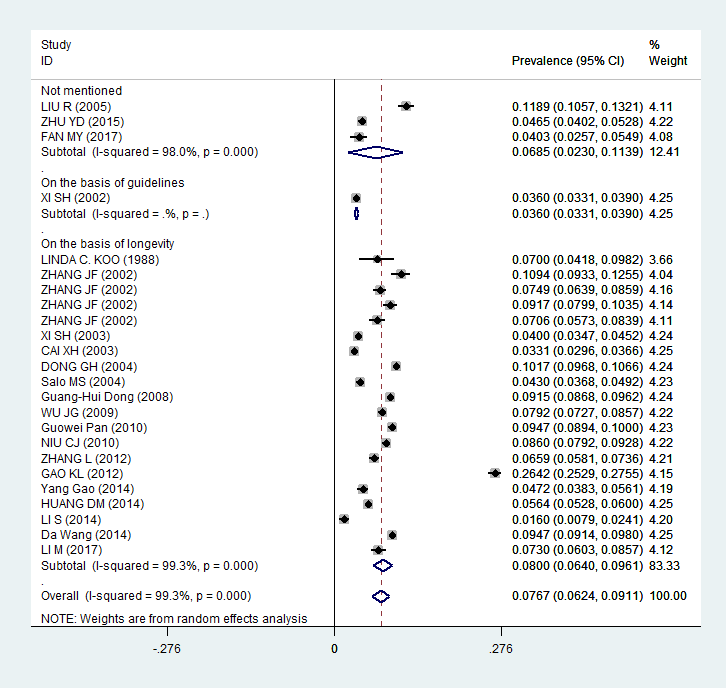


**Figure S16** Pooled chronic cough prevalence of children stratified by quality of articles assessed by AHRQ. Abbreviations: CI, confidence intervals. NOTE: The four author labels of ZHANG JF 2002 are from the same literature.


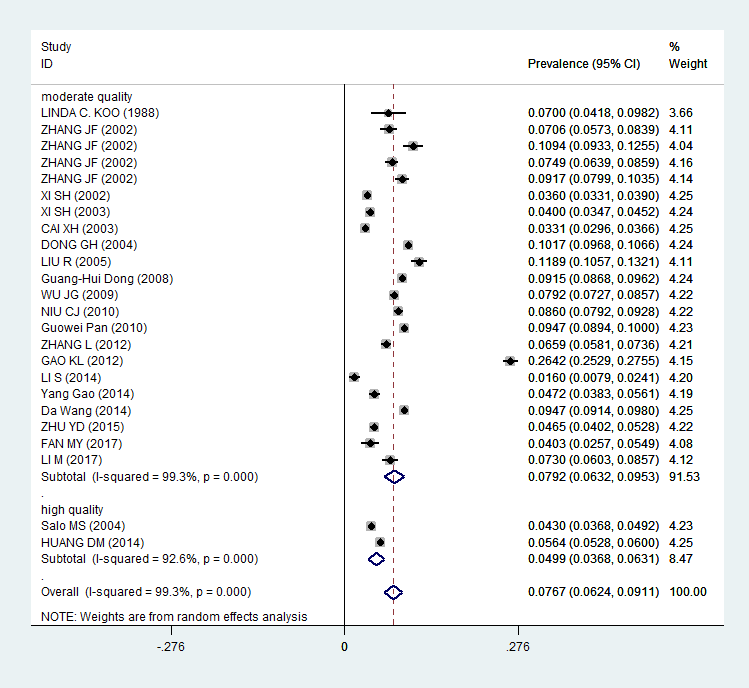


**Figure S17** Pooled chronic cough prevalence of children stratified by prevalence definitions. Abbreviations: CI, confidence intervals. NOTE: The four author labels of ZHANG JF 2002 are from the same literature.


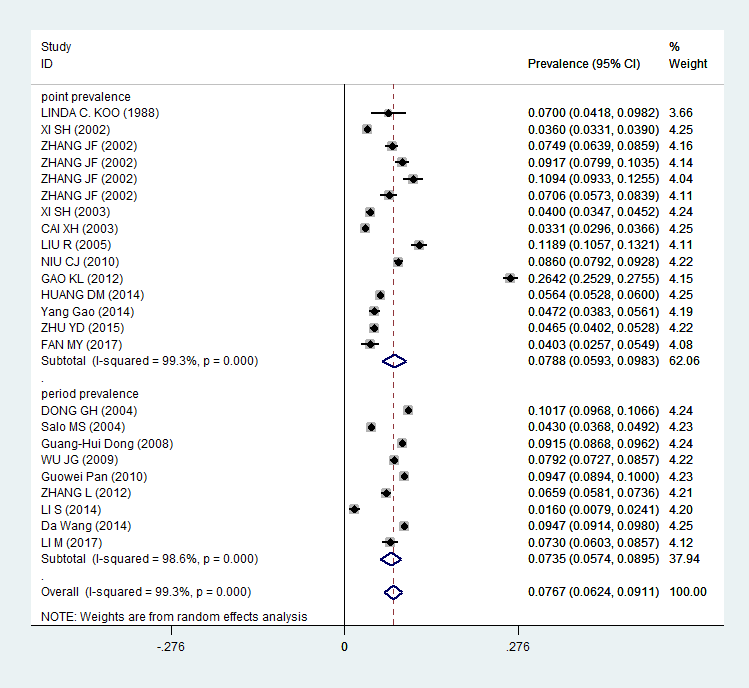


**Figure S18** Funnel plot for prevalence in studies of adults for chronic cough.


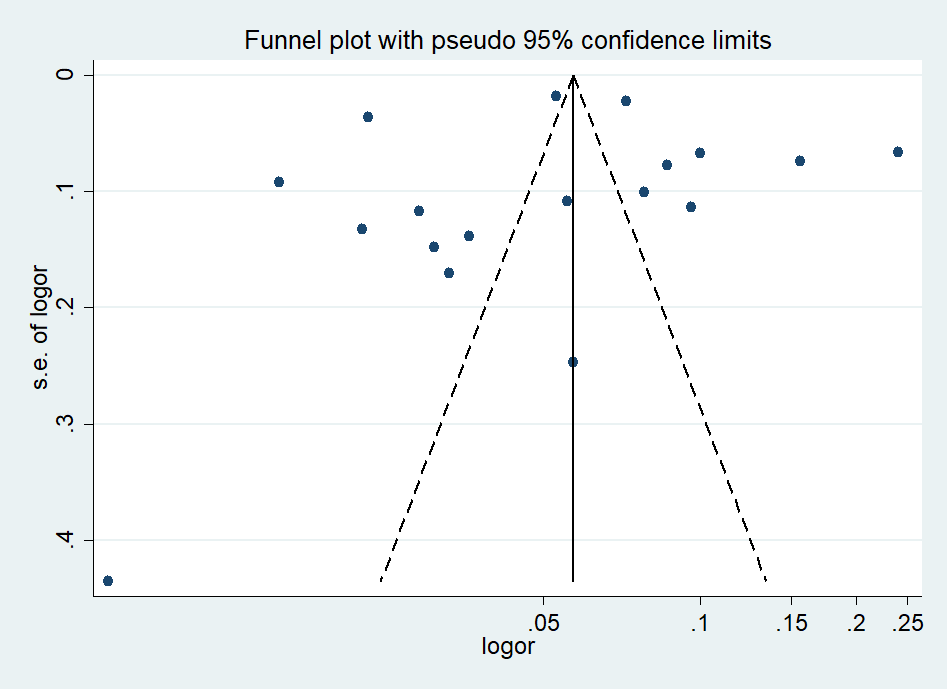


**Figure S19** Sensitivity analysis for prevalence in studies of adults for chronic cough. Abbreviations: CI, confidence intervals. NOTE: The three author labels of ZHANG JF 1999 are from the same literature, and the two author labels of Venners 2001 are from the same literature.


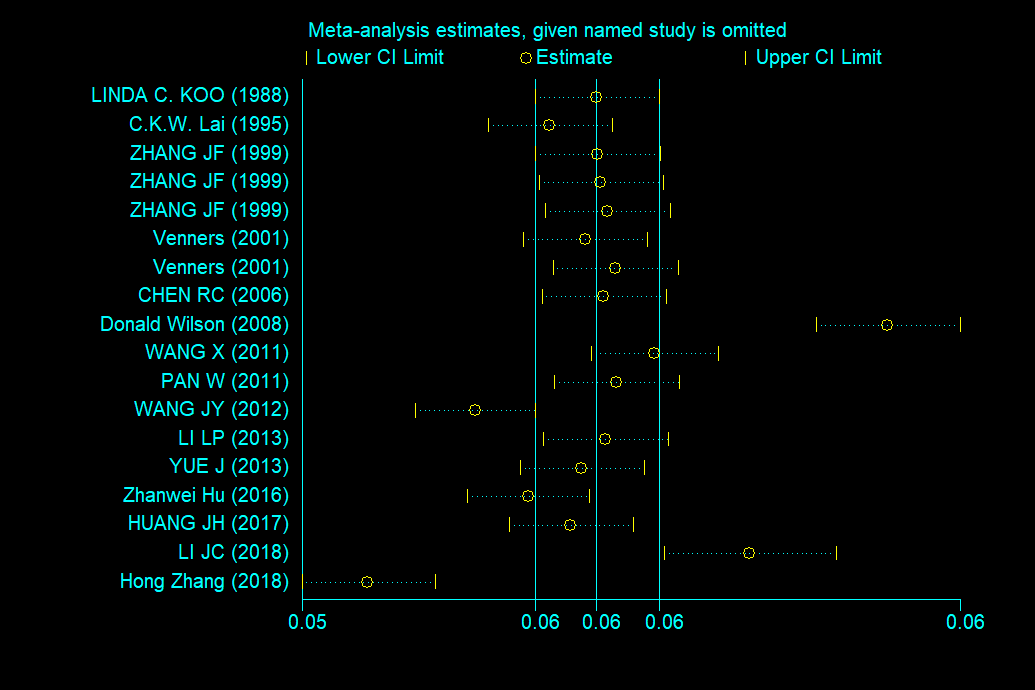


**Figure S20** The prevalence of chronic cough in adults after exclusion of the nationwide study (Li JC 2018). Abbreviations: CI, confidence intervals. NOTE: The three author labels of ZHANG JF 1999 are from the same literature, and the two author labels of Venners 2001 are from the same literature.


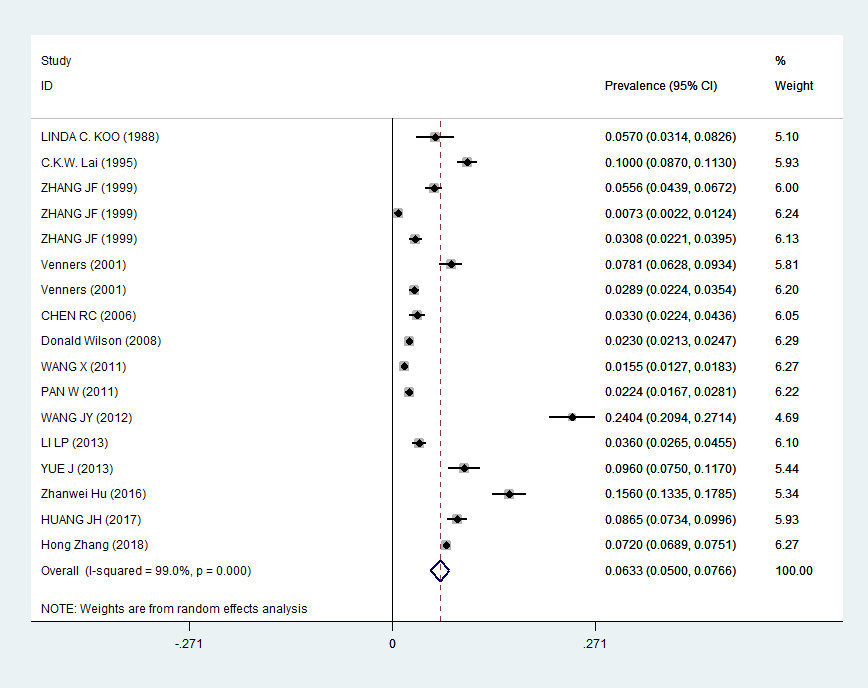


**Figure S21** The prevalence of chronic cough in adults after exclusion of the low prevalence study (ZHANG JF 1999). Abbreviations: CI, confidence intervals. NOTE: The two author labels of ZHANG JF 1999 are from the same literature, and the two author labels of Venners 2001 are from the same literature.


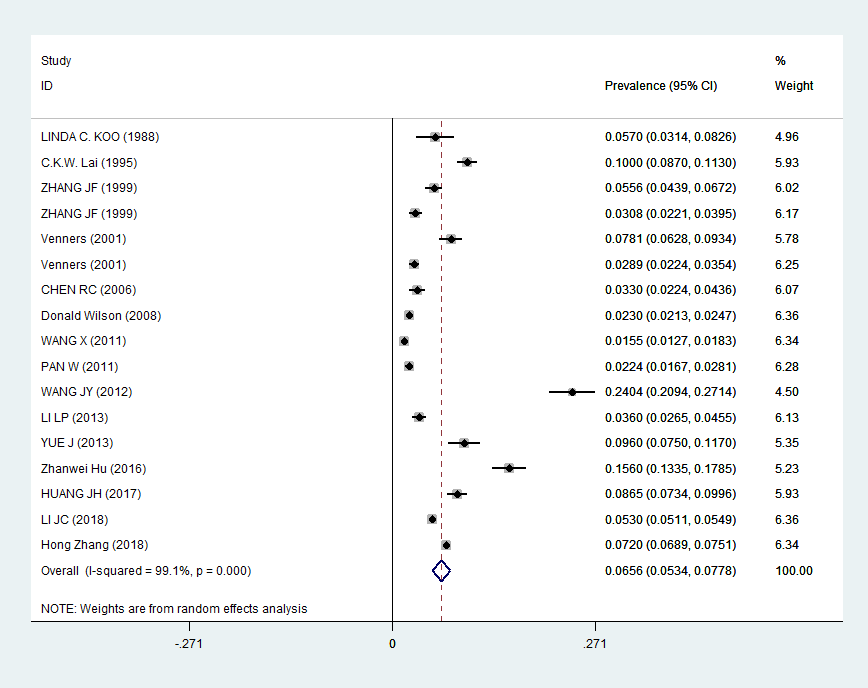


**Figure S22** Funnel plot for prevalence in studies of children for chronic cough.


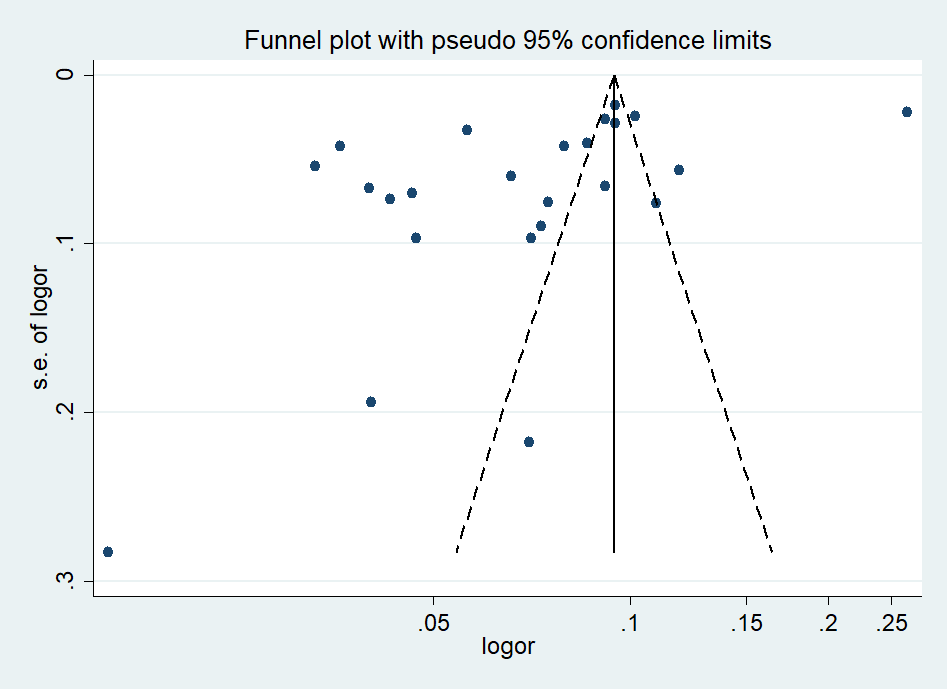


**Figure S23** Sensitivity analysis for prevalence in studies of children for chronic cough. Abbreviations: CI, confidence intervals. NOTE: The four author labels of ZHANG JF 2002 are from the same literature.


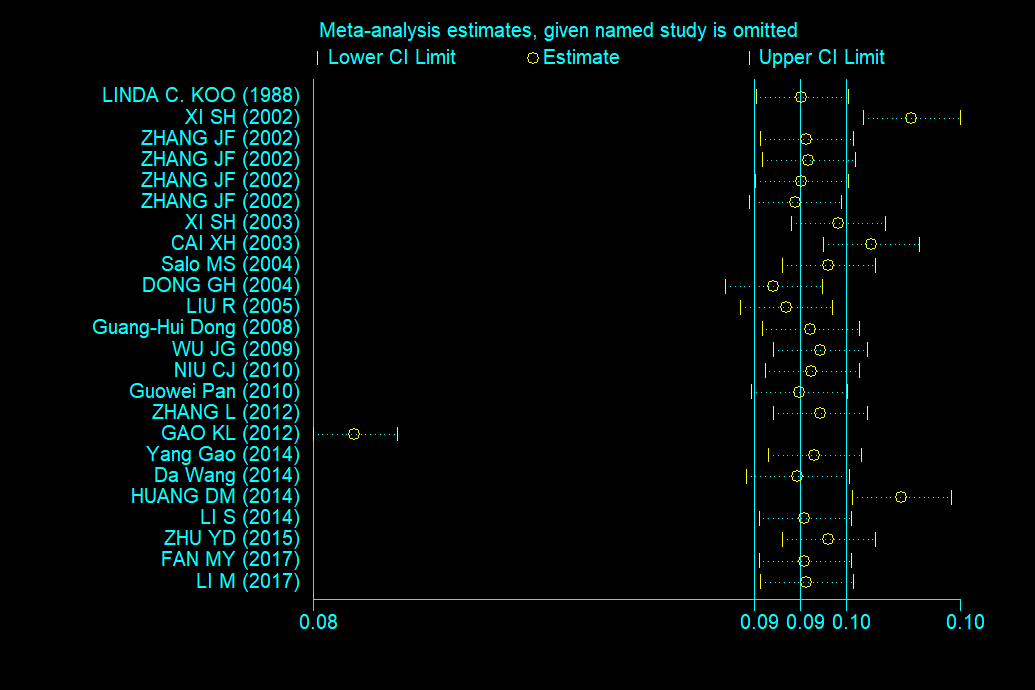


**Figure S24** Pooled prevalence of chronic cough in China (including adults and children). Abbreviations: CI, confidence intervals. NOTE: The three author labels of ZHANG JF 1999 are from the same literature, the two author labels of Venners 2001 are from the same literature, and the four author labels of ZHANG JF 2002 are from the same literature.


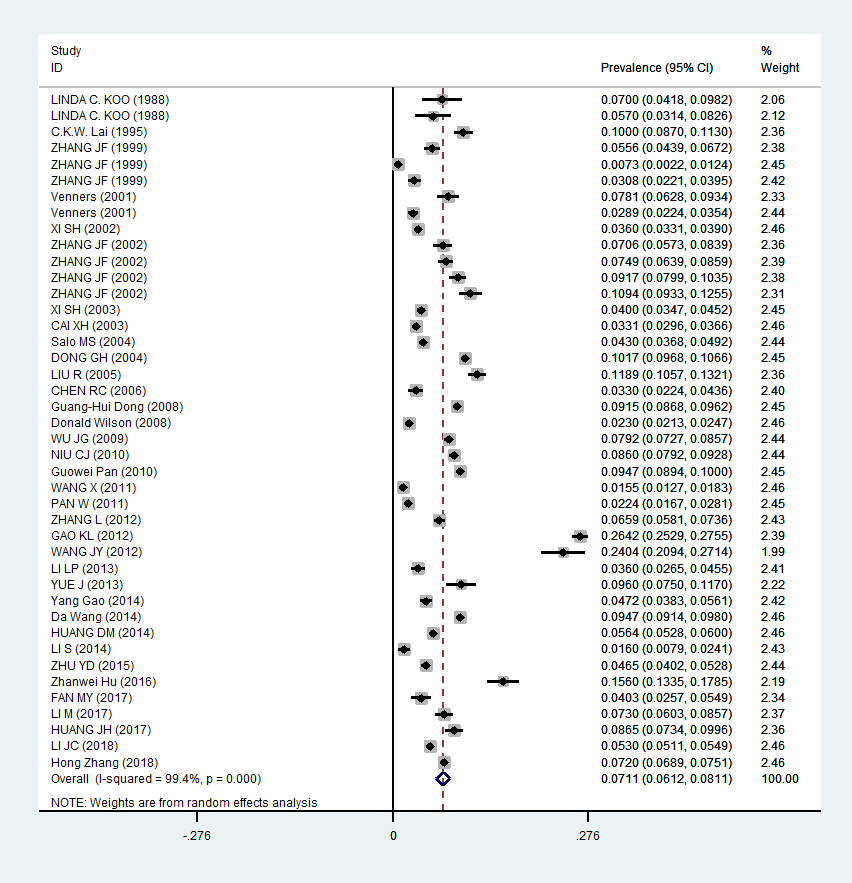

Supplement: Supplementary file 6 — Additional file 6. Fig. S1. Distribution of children with chronic cough across Mainland China. NOTE: Red star in the map represents Beijing City. The map was developed in XL Toolbox NG by ourselves, without the conflict of copyright. Fig. S2. Pooled chronic cough prevalence of adults stratified by region. Abbreviations: CI, confidence intervals. NOTE: The three author labels of ZHANG JF 1999 are from the same literature, and the two author labels of Venners 2001 are from the same literature. Fig. S3. Pooled chronic cough prevalence of adults stratified by diagnostic criteria. Abbreviations: CI, confidence intervals. NOTE: The three author labels of ZHANG JF 1999 are from the same literature, and the two author labels of Venners 2001 are from the same literature. Fig. S4. Pooled chronic cough prevalence of adults stratified by year of publication. Abbreviations: CI, confidence intervals. NOTE: The three author labels of ZHANG JF 1999 are from the same literature, and the two author labels of Venners 2001 are from the same literature. Fig. S5. Pooled chronic cough prevalence of adults stratified by age. Abbreviations: CI, confidence intervals. NOTE: The three author labels of ZHANG JF 1999 are from the same literature, and the two author labels of Venners 2001 are from the same literature. Fig. S6. Pooled chronic cough prevalence of adults stratified by sampling methods. Abbreviations: CI, confidence intervals. NOTE: The three author labels of ZHANG JF 1999 are from the same literature, and the two author labels of Venners 2001 are from the same literature. Fig. S7. Pooled chronic cough prevalence of adults stratified by sample size. Abbreviations: CI, confidence intervals; ES, Effect Size. NOTE: The three author labels of ZHANG JF 1999 are from the same literature, and the two author labels of Venners 2001 are from the same literature. Fig. S8. Pooled chronic cough prevalence of adults stratified by prevalence definitions. Abbreviations: CI, confidence intervals; ES, [file 12890_2022_1847_MOESM6_ESM.docx]
